# The first report on detecting SARS-CoV-2 inside bacteria of  the human gut microbiome: A case series on asymptomatic family members and a child with COVID-19

**DOI:** 10.12688/f1000research.77421.3

**Published:** 2024-10-16

**Authors:** Carlo Brogna, Simone Cristoni, Mauro Petrillo, Domenico Rocco Bisaccia, Francesco Lauritano, Luigi Montano, Marina Prisco, Marina Piscopo

**Affiliations:** 1 Department of Microbiology, Craniomed group Srl, Montemiletto, Avellino, 83038, Italy; 2 Isb Srl. Ion source & Biotechnologies S.r.l,, Bresso, Milano, 20091, Italy; 3 European Commission, Joint Research Centre (JRC), Ispra, Italy; 4 Seidor Italy S.r.l., Milano, 20129, Italy; 5 Andrology Unit and Service of LifeStyle Medicine in Uro-Andrology, Local Health Authority (ASL), Salerno, 84124, Italy; 6 Department of Biology, University of Naples Federico II, Napoli, 80126, Italy

**Keywords:** SARS-CoV-2, gut microbiota, bacteriophage, feces, diarrhea, nasopharyngeal swab, fecal oral transmission, TEM image, case series

## Abstract

Many studies report the importance of using feces as source sample for detecting SARS-CoV-2 in patients with COVID-19 symptoms but who are negative to oropharyngeal/ nasopharyngeal tests. Here, we report the case of an asymptomatic child whose family members had negative results with the rapid antigen nasopharyngeal swab tests. The 21-month-old child presented with fever, diarrhea, bilateral conjunctivitis, and conspicuous lacrimation. In this study, analysis for the presence of SARS-CoV-2 in fecal samples by using Luminex technology allowed accurate detection of the presence of the viral RNA in the feces of the child and of all her relatives, which thus resulted to be positive but asymptomatic. It is the first time that SARS-CoV-2- is observed inside the bacteria of the human gut microbiome and outside a matrix resembling extracellular bacterial lysates, in agreement with a bacteriophage mechanism with the images obtained by transmission electron microscopy (TEM), post-embedding immunogold, and by fluorescence microscope. In addition to the typical observations of respiratory symptoms, accurate evaluation of clinical gastrointestinal and neurological symptoms, combined with efficient highly sensitive molecular testing on feces, represent an efficient approach for detecting SARS-CoV-2, and for providing the correct therapy in challenging COVID-19 cases, like the one here reported.

## Introduction

In the past two years, humanity has been combating the severe acute respiratory syndrome coronavirus 2 (SARS-CoV-2). SARS-CoV-2 is a positive, single-stranded RNA virus of the *Coronaviridae* family, specifically of the subfamily *Orthocoronavirinae* (usually called “coronaviruses”). Its closest known relatives are those found in bat feces, like the coronavirus RaTG13. ^1^ Xu *et al*. (2020) ^2^ studied viral behavior in 10 children, ranging in age from two months to 15 years. Although all of them were positive to the initial nasopharyngeal test, for eight of them, the viral charge was also positive in the stool. Moreover, they continued to test positive in the stool even after the negative nasal swab for several days after hospital discharge. In another Chinese study, the researchers found viral positivity in the fecal samples of 205 patients. ^4^ Many studies ^3^^–^^5^ have observed that fecal-oral transmission of the virus is possible and that it is very common to detect this virus in feces. Nevertheless, in comparison to the closest SARS-like viruses, SARS-CoV-2 appears to diverge in the receptor-binding domain of the spike glycoprotein, which is considered a key player in the entrance of the virus in human eukaryotic cells throughout its interaction with the angiotensin-converting enzyme 2 receptor (ACE-2), which in turn is considered the entry point of the virus. ^6^ ACE-2 receptors and host cell transmembrane serine protease 2 (TMPRSS2) are abundant throughout the intestinal tract ^7^^,^^8^ and several studies have reported altered intestinal bacterial flora or intestinal bacterial co-infection in COVID-19 patients. ^9^^–^^11^ In terms of hosts, coronaviridae members are neither human-specific nor new in terms of discovery and treatments: a recent review describes the numerous zoonoses caused by the Coronaviridae family members, ^12^ and scientists searched for the pathogen in the stool, ^13^ a method that was, and continues to be, very common in the veterinary field. Among the coronaviruses previously found and analyzed in feces, there are those responsible for animal diseases like the calves' enzootic pneumonia (caused by Bovine coronavirus, BCoV), or the porcine epidemic diarrhea (caused by the Porcine Epidemic Diarrhea Virus, PEDV). These diseases and other coronavirus-related ones very often show as initial clinical manifestation of violent diarrhea, and the affected animals have a significant alteration of the intestinal mucosa. ^12^^,^^14^^,^^15^ Observations of possible links between the animal gut microbial environment and coronaviruses have been reported in some studies, ^16^^–^^20^ supported also by the use of transmission electron microscopy (TEM) image analysis which screens and looks for viruses-like particles. ^8^^,^^21^ The observation of SARS-CoV-2 particles by TEM can complement the molecular traces of it. ^22^ Finally, it is worth noting that almost all of the latest characterized SARS-like viruses have been found and sequenced in bat fecal samples. ^23^

Here, we report the case of a symptomatic child whose family members had negative results with rapid antigen nasopharyngeal swab test. Analyses of fecal samples detect the viral RNA presence in the feces of the child and of all her relatives, which thus resulted to be positive asymptomatic. Microscope image analyses confirm the presence of SARS-CoV-2-like particles on fecal samples of the family and suggest that bacteria, reservoirs of the virus, are the most critical factors of fecal-oral transmission in this pandemic. The present case report also emphasizes the importance of the rapid detection of SARS-CoV-2 in symptomatic and non-symptomatic subjects with negative results from nasal and oropharyngeal swabs by analyzing stool samples and emphasizes the importance of the bacteriophagic mechanism of the virus and its fecal-oral transmission.

## Case series description

A 21-month-old female, Caucasian child, presented to us with severe bilateral conjunctivitis, conspicuous lacrimation, diarrhea, malodorous stools, restlessness, and fever (38°C). Conjunctivitis and lacrimation are known to be common symptoms in children affected by coronavirus in general and also by SARS-CoV-2. ^24^^–^^29^ Recently it was observed also that the “Arcturus” variant of SARS-CoV-2 is also particularly impactful with these two symptoms in children ( https://www.cnbctv18.com/healthcare/covid-new-symptom-variant-arcturus-omicron-conjunctivitis-itchy-eyes-children-16399051.htm). The child’s medical history was negative for any disease. Parents reported that about a year earlier, she had a period when she had a severe cold. They were alarmed by violent diarrhea, which was preceded by 24 hours of constipation, as well as by abnormal bilateral conjunctivitis with uncontrollable lacrimation. Rapid blood tests showed the following values (in bold are those out of normal range, NR): creatinine 0.18 mg/dL (NR: 0.40-1.10 mg/dL); glucose 97 mg/dL (NR: 60-110 mg/dL); aspartate transaminase 45 I.U. (NR: 10-50 I.U.); alanine transaminase 28 I.U. (NR: 10-35 I.U.); sodium 139 mEq/L (NR: 136-150 mEq/L); potassium 5.82 mEq/L (NR: 3.50-5.10 mEq/L); chloride 95 mEq/L (NR: 98-107 mEq/L); calcium 5.50 mEq/L (NR: 4.25-5.25 mEq/L); C-reactive protein 2.60 mg/L (NR: 0-5 mg/L); iron 28 mcg/dL (NR: 59-158 mcg/dL). Other complete blood count values were in the normal range.

**Figure 1.**
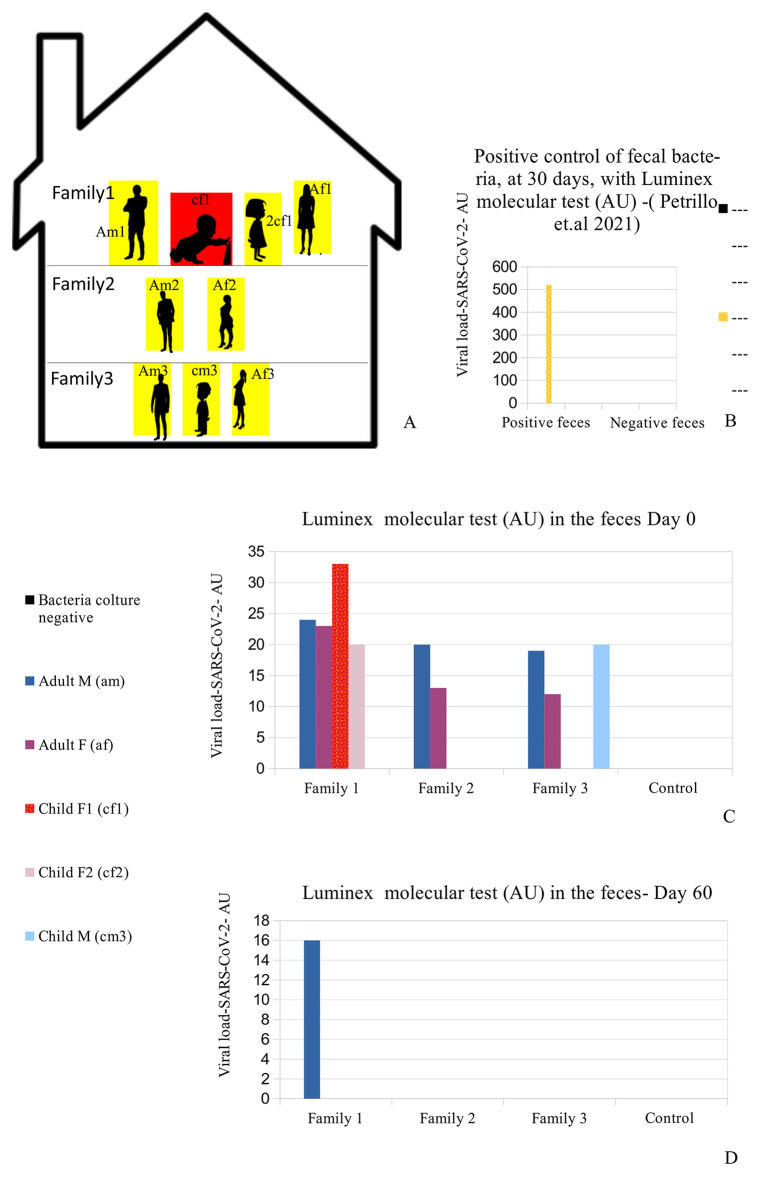
Case presentation and RNA viral concentration. (A) Distribution of the nine people analyzed in the family. Red (cf1: child female family 1) shows the child who was symptomatic and had positive results on the fecal molecular test. Yellow: the other family 1,2,3 members (Am1: adult male family 1; af1: adult female family 1; 2cf1: 2nd child female family 1; am2: grandfather family 2; af2: grandmother family 2; am3: adult male family 3; af3: adult female family 3; cm3: child male family 3) who had positive results on the Luminex molecular fecal test but negative results on the rapid antigen nasopharyngeal swab test. (B) This is the positive control of patient am1's bacteria, derived from a stool sample, after 30 days of bacterial culture using our previously published method, ^30^ performed with the Luminex molecular assay. The molecular assay reported a viral RNA concentration growth of up to 520 AU (arbitrary unit). (C-D) RNA Viral concentration initially and after 60 days. The family members hired supplemental therapy, only as re-balancers of bacterial flora, with colloidal copper and bromelain, as well as with probiotics therapy, only as re-balancers of bacterial flora, with *Lactobacillus reuteri* and *Bacillus clausii.*

The Caucasian family (six adults, and three children) came to us, in the autumn of 2020, during one of the Italian regional lockdown periods. Some specific information on the family members were recorded, including age, sex, medical history, occupations, and relationships (see Table 1). They live in close proximity, divided among three apartments in one building ( Figure 1 panel A). The parents reported that the children never had a babysitter since this task was entrusted to their grandparents, who were in their building. Moreover, they reported that since the outbreak of the pandemic (March 2020), they had adopted a series of measures, probably excessive in their opinion, with the purpose of protecting the grandparents and children from sickness. Such measures included no contact with people outside the family context, disinfection of every product purchased, no summer holidays, no eating at restaurants or other public places, and limited outings for the four parents (am1, af1, am3, af3) for work reasons only. The grandfather (am2), grandmother (af2), and the three children (cf1, 2cf1, cm3) did not leave the building for the duration of the lockdown ( Figure 1A and Table 1). All the parents (am1, af1, am3, af3) of the children working in the health care sub-area left home daily to work, and one of them worked in another geographical region. Considering their work position, it is most likely that the family infection started with the contagiousness of one of the four parents (am1, af1, am3, af3) who were asymptomatic during working hours. Of interests is the medical history of one adult (am1), the father of child cf1 (our COVID-19 patient), that was hospitalized precisely one year prior (autumn 2019) with escalating symptoms of violent diarrhea, abdominal pain, fever (38°C), dyspnea, cough, headache, shortness of breath, and fainting. There was saturation of 91 SpO2%, right bundle branch block, increased D-Dimer, increased liver values (GOT and GPT), and mild lymphopenia, treated with antibiotics.

**Table 1.** Information about family members.

Subject	Symptoms	Rapid antigen nasopharyngeal swab test (COVID-19 Ag Rapid Test Device, Abbott 41FK10	Stools Luminex AU Test before therapy (Initial)	Stools Luminex AU Test 60 days after therapy (Final)	Medical history or comorbidities	Age	Degree of the kinship with the child (cf1) affected by Covid-19	Occupation
am1	No	Negative	24	16	Hepatic Steatosis	43	Father	Worker in the health care sub-area
af1	No	Negative	23	0	Congenital heart disease: patent foramen ovale (PFO) and atrial septal aneurysm (ASA)	38	Mother	Worker in the health care sub-area
cf1	Yes: bilateral conjunctivitis, conspicuous lacrimation, diarrhea, restlessness, and fever (38°C)	Negative	33	0	Healthy	21 mounths	the child affected by COVID-19	–
2cf1	No	Negative	20	0	Healthy	8	Sister	Primary school student- lessons in "didactics distance“ (DAD) for the lookdown period.
am2	No	Negative	20	0	Benign prostatic hyperplasia (BPH)	69	maternal grandfather	Retired- full-time grandfather with grandson cf1, cf2 and cm3
af2	No	Negative	13	0	Diverticulosis; arterial hypertension	67	maternal grandmother	Retired- full-time grandfather with grandson cf1, cf2 and cm3
am3	No	Negative	19	0	Healthy	42	Uncle	Worker in the health care sub-area
af3	No	Negative	12	0	Healthy	39	Maternal aunt	–
am3	No	Negative	20	0	Healthy	2	Cousin	–

Am1: adult male family 1; af1: adult female family 1; cf1: child female family 1; 2cf1: 2 ^nd^ child female family 1; am2: grandfather family 2; af2: grandmother family2; am3: adult male family 3; af3: adult female family 3; cm3: child male family 3.

We initially performed rapid antigen nasopharyngeal swab test (COVID-19 Ag Rapid Test Device, Abbott 41FK10) on the child (cf1), and it was negative. The same test was also performed on the parents (am1, af1) and the other six family members, and all results were negative. We had, in line with previous studies, ^31^^,^^32^ experience of multiple negative results SARS-CoV-2 real-time reverse transcriptase polymerase chain reaction (RT-PCR) tests on oropharyngeal/nasopharyngeal (OP/NP) swab samples from individuals with a strong clinical suspicion of COVID-19. ^33^ Being in the presence of a very young patient, it was decided to adopt a fast high-throughput COVID-19 screening approach to detect the presence of SARS-CoV-2 directly from stool samples: in the following 24 hours, stool samples were collected from all nine family members, and molecular testing for SARS-CoV-2 was performed by using Luminex technology ^34^^,^^35^ as described by us previously. ^30^ Negative and positive controls as bacterial cell cultures of stool samples were those used and described in this previous study. ^30^

A summary of the analyses is reported (all methods and materials are detailed in supplementary materials s.m.) in Figure 1C-D and Table 1: all family members had positive results to the Luminex molecular test, and the child with symptoms (cf1) showed the highest value of the Luminex assay. The other family members did not manifest any symptoms, despite being positive for the presence of viral RNA in their stools.

**Figure 2.**
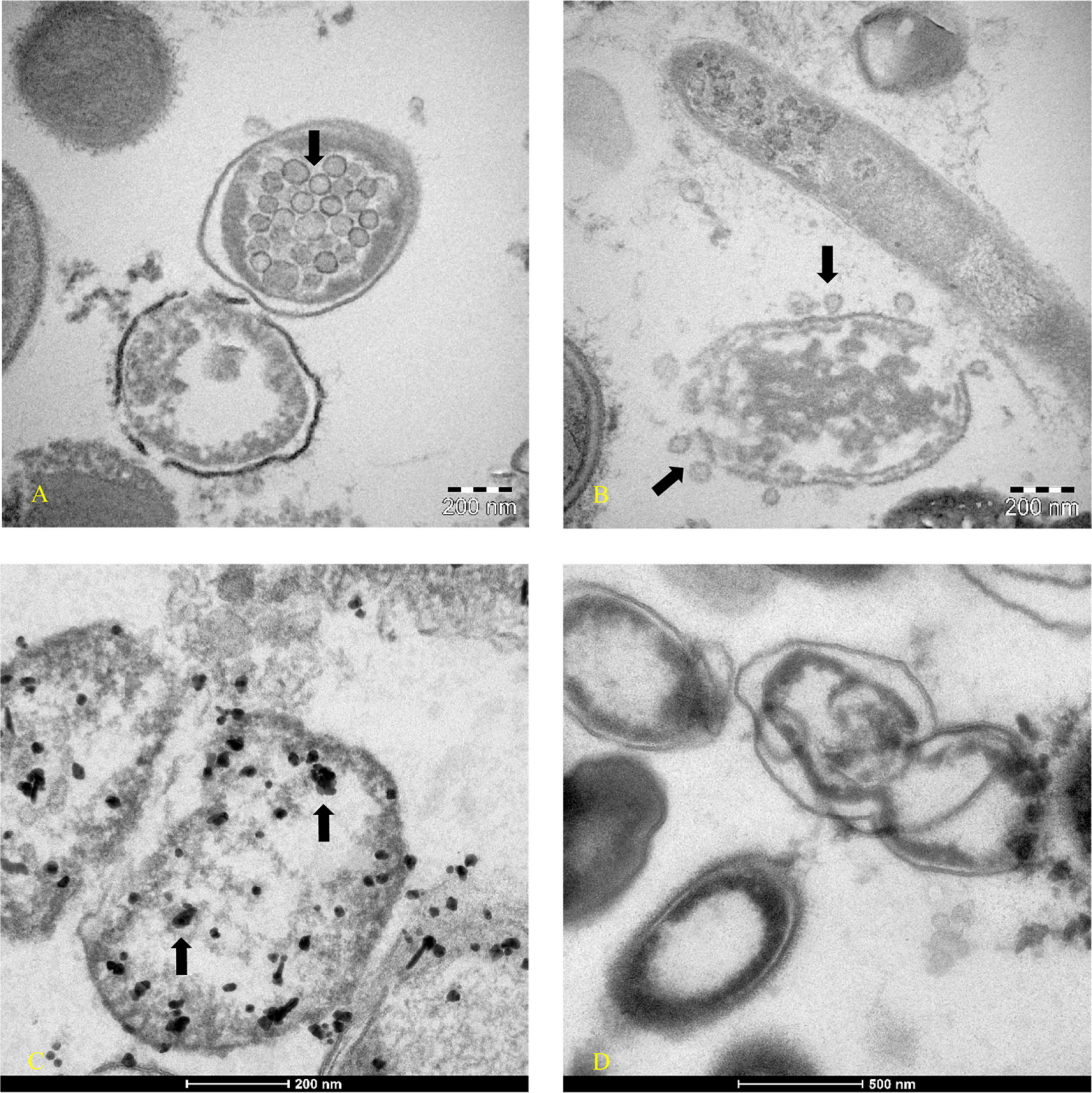
Transmission electron microscopy (post-embedded immunogold). Images were obtained at day 30 of bacterial culture of patient am1’s feces, in which a molecular test with Luminex confirmed the presence of SARS-CoV-2 and an increase of RNA viral concentration from the initial 24 arbitrary unit (AU) to 520 AU final. (A-B) Transmission electron microscope images (panels A and B -TEM FEI- Thermo Fisher Tecnai G2 operating at 120 kV) show SARS-CoV-2 (indicated by black arrows) inside a bacterium (A) and outside a matrix resembling extracellular lysate of a bacterium (B). (C-D) Post-embedding immunogold: rabbit monoclonal to SARS-CoV-2 Nucleocapsid protein antibodies ligating to the secondary anti-rabbit antibody 10nm gold-conjugated indicated the virus inside bacteria of gut microbiota (Tecnai G2 Spirit BioTwin; FEI equipped with a VELETTA CCD digital camera (Soft Imaging Systems GmbH)). (D) Negative control of bacterial stool culture of a healthy person after 30 days, without primary antibody with only the secondary antibody. Note: Orenstein et al. ^36^^,^^37^ point out that coronavirus particles can be visible at different densities (particularly ribonucleoprotein). The authors’ state: “ *Mature particles had a spherical central core, which was clear, contained electron-dense granules (nucleocapsid) or was totally electron-dense (… …)”.* The viral particles highlighting coronaviruses in reference 37 are identical to those shown in our Figures 2A and 2B. In addition, in our Figure 2, it is possible to visualize the viral particles at different densities from the left side to the right side within the bacterium.

The child was treated for 48 hours only with rehydration and probiotics only; because of the absence of significant symptoms such as cough or dyspnea, no cortisone or antibiotics were administered. Conjunctivitis and lacrimation ceased about 72 hours later and the patient was discharged. The entire family, including the reported patient, were then instructed to take probiotics ( *Lactobacillus reuteri,* 100 million units, one time per day, and *Bacillus clausii* 2 billion units, per day) in addition to bromelain, 300 mgr. per day, and colloidal copper, 20 ppm (parts per million) per day for 30 days, only as re-balancers of bacterial flora. After 60 days, both the rapid antigen nasopharyngeal swab test (COVID-19 Ag Rapid Test Device, Abbot 41FK10) and the Luminex test were repeated: all family members were negative to the rapid antigen tests, and only one family member ( Figure 1D -am1) continued to have Luminex positive results. Patient am1, male, Caucasian, and a healthcare employee, continued the treatment until he became negative at day 90 for the presence of SARS-CoV-2 in stools. The feces of this patient was cultured in bacterial culture media and after 30 days, the pellet of bacteria, have been analyzed by TEM, immune-EM, and by fluorescence microscopy, and a set of obtained images is shown in Figure 2 (for more details see supplementary material-s.m.) (see the paragraph in the end of the manuscript). At day 30 of bacterial culture of feces patient am1, the Luminex molecular test confirmed the presence of SARS-CoV-2, and the RNA viral concentration was increased from 24 arbitrary unit (AU) (initial) to 520 AU (Final) ( Figure 1B) in accordance with our previous observations. ^30^ Transmission electron microscope images (panels A and B of Figure 2-Tecnai G2 Spirit BioTwin; FEI, equipped with a VELETTA CCD digital camera -Soft Imaging Systems GmbH) SARS-CoV-2 (black arrows) inside a bacterium (A) and outside a matrix resembling extracellular lysate of a bacterium (B). No eukaryotic cells have been ever observed after 30 days of bacterial culture. Post-embedding immunogold ( Figure 2 Panel C, D): bacteria pellets were fixed with a mixture of 0.05% glutaraldehyde of 4% paraformaldehyde in 0.1M PBS (Phosphate-buffered saline) buffer, washed in PBS buffer, pelleted at 10000g and included in 3% agarose. The agarose block was cut into tissue-size pieces and the slices were post-fixed in 2% OsO _4_, dehydrated in a series of ethanol solutions of increasing concentration and in propylene oxide and finally embedded in Epon 812. Thin sections were cut from embedded specimens using Reichert Jung Ultra microtome and are applied to Formvar/Carbon Supported nickel grids. Sections were blocked with normal goat serum for 1h at room temperature, incubated with rabbit monoclonal to SARS-CoV-2 nucleocapsid protein antibody (EPR24334-118, Abcam) and then with secondary anti-rabbit antibody 10nm gold-conjugated (Aurion). Electron microscopy images were acquired from thin sections under an electron microscope (Tecnai G2 Spirit BioTwin; FEI) equipped with a VELETTA CCD digital camera (Soft Imaging Systems GmbH). More details are presented in reference 36 e in supplementary materials (see the paragraph at the end of the manuscript).

**Figure 3.**
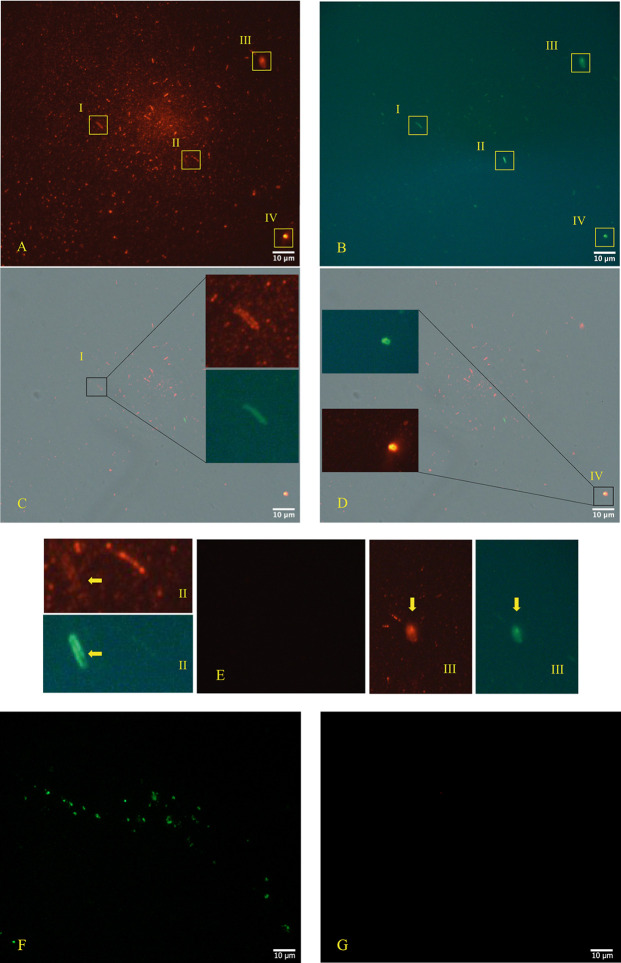
Fluorescence microscope images. Panels A, B, C, D (Zeiss Axioplan 2, Axiocam 305 color, magnification 100×) show immunofluorescence staining versus SARS-CoV-2 nucleocapsid protein (red light), gram positive bacteria (green light). Panel E is the negative control and panels F and G show a group of gram+ bacteria by fluorescence, derived from the stool bacteria culture of a healthy 18-month-old child (with healthy parents and never ill with SARS-CoV-2 at the time of collection and with and with their written consent) negative to molecular test to SARS-CoV-2, although the other primary antibody to the nucleocapsid protein is also included and does not show a red signa. The roman numerals I,II,III,IV and yellow rectangles indicate four gram-positive bacteria (green light) infected by SARS-CoV-2 (red light). Note: *the Rockland primary antibody (Sars Nucleocapsid Protein Antibody [Rabbit Polyclonal]—500 μg 200-401-A50 Rockland) we used for immunofluorescence experiments has no less than 76 references where it has been used, and its remarkable specificity towards SARS-CoV-2 was defined. The references can be seen on the following page:* *https://www.rockland.com/catego-ries/primary-antibodies/sars-nucleocapsid-protein-antibody-200-401-A50/#productReferenceSectionWrapper* *(accessed on 20 February 2024) and the Gram-positive bacteria antibody [BDI380] Genetex is a mouse monoclonal antibody that has been validated and tested to be reactive toward many Gram-positive bacteria—precisely what we were interested in finding out. The specifications can be found at the following link:* *https://www.genetex.com/Product/Detail/Gram-Positive-Bacteria-antibody-BDI380/GTX42630* *(accessed on 20 February 2024). The authors stated the following: “Reactive with lipoteichoic acid (LTA) of many Gram-positive bacteria. Cross-reacts with Listeria monocytogenes (all serotypes), Streptococcus pneumoniae, Staphylococcus aureus, Staphylococcus epidermidis, Enterococcus faecium, Bacillus cereus, Bacillus subtilis and group B Streptococcus (weak). Does not react with Clostridium perfringens”.*

The immunofluorescence microscope ( Figure 3, panels A; B, C, D - Zeiss Axioplan 2, Axiocam 305 color, magnification 100×) was performed in according to manufactures’ protocol, ^38^^,^^39^ using as primary antibodies versus SARS-CoV-2 Nucleocapsid protein (“Sars Nucleocapsid Protein Antibody [Rabbit Polyclonal] - 500 μg 200-401-A50 Rockland”, and the “Goat anti-Rabbit IgG (H+L) Cross-Adsorbed Secondary Antibody, Cyanine3 #A10520” as secondary antibody). It was used also a primary antibody versus gram-positive bacteria (“Gram-Positive Bacteria Ab (BDI380), GTX42630 Gene Tex”) and “Goat anti-Mouse IgG (H+L), Super-clonal™ Recombinant Secondary Antibody, Alexa Fluor 488” as secondary antibody. The images confirm the presence of SARS-CoV-2 particles (red light in the fluorescence images) in relationship with the bacteria (green light in the fluorescence images).

To our knowledge, this is the first time that a member of coronaviruses’ family, the SARS-CoV-2, has been observed inside the bacteria of the human gut microbiome ( Figure 2 panel A – Figure 2 panel C) and outside a matrix resembling extracellular bacterial lysates ( Figure 2, panel B), in agreement with a phage-like behavior reported by us. ^21^

## Discussion

Zheng F. *et al*. ^40, 41^ observed that gastrointestinal symptoms are common in children with SARS-CoV-2 and are associated with fever, nausea, vomiting, and abdominal pain. However, their case series is probably not very large both because it is known that more than half of sick children have mild to moderate symptoms and because hospitalizations are not as common as for other respiratory viruses. ^40^ A recent example of the possibility of fecal-oral transmission is well described in a short communication by Hansen *et al*. ^42^ These authors reported the case of an 86-year-old man who, despite of having been vaccinated (first dose of BNT162b2 mRNA COVID-19 vaccine), eighteen days after vaccination was admitted to the hospital for diarrhea, with no other symptoms of COVID-19, and had negative results on antigen and PCR testing until day 26, when he died of acute renal and respiratory failure. On day 24, the older man's roommate tested positive for SARS-CoV-2 RT-PCR on a nasal swab. Autopsy results of the 86-year-old decedent indicated the presence of the virus in the organs examined except for the liver and olfactory bulb.

In one of the first studies on SARS-CoV-2 in Wuhan, prominent symptoms of COVID-19 patients are described, including diarrhea ^43^ and in children, gastrointestinal disorders are the most prevalent. ^40^ The persistence of coronaviruses in feces, for a long time, had already been observed many years ago. In one of the first case reports of 1982, Baker *et al*. ^44^ described the case of a 47-year-old Indian man who underwent surgery for a duodenal ulcer when he was 13 years old. The symptoms that forced hospitalization were diarrhea and steatorrhea. The man was monitored for eight months, and in 17 fecal samples, coronavirus-like particles were observed by electron microscopy. The images show two ovoid/geoid shaped coronavirus particles with the spike protein evident and one circular shaped coronavirus particle but without surface proteins, like those here reported in Figure 2.

Inclusion of symptoms other than respiratory, such as gastrointestinal symptoms, seems to be very important in the diagnostic process. Although diarrhea and conjunctivitis with lacrimation, as in our case, may be unlinked, they can be related to each other if the gut microbiota and the central, peripheral, and autonomous nervous systems are taken into account. Also with other Coronavirus NL63 was present the same symptoms. ^67^ The gut microbiota ^45^ seems to be extremely important and interconnected with the central, peripheral, autonomic, neuroimmune, and neuroendocrine nervous system axis. An altered gut microbiota or the total absence of bacteria, as in germ-free mice, can affect areas of the brain, including the hippocampus, the point of end of olfactory system. ^46^ Several studies have reported an impairment of intestinal gut microbiota ^47^ or respiratory and intestinal bacterial coinfection in COVID-19. ^48^

As shown in Figures 2- 3, bacteria could play crucial role in the possibility of fecal-oral transmission. This news isn’t so far away from the most recent studies ^30^ in which we described that RNA replication of the SARS-CoV-2 virus can take place in bacterial cultures. We also described that the use of antibiotics can decrease its replication *in vitro.* Moreover, in the same work, we observed, by mass spectrometry, the mutational phenomenon of viral proteins in bacterial cultures. Other authors have also noted the possibility that the spike protein of the SARS-CoV-2 may interact with the lipopolysaccharide of *Escherichia coli* ^49^ or that the absence of proteobacteria could play a key role in the pathogenesis of respiratory viral diseases. ^46^ This is why early localization in the stool assumes considerable importance. Since the discovery of SARS-CoV-2, a plethora of commercial tests have become available, and, currently, more than 1,700 tests are commercialized in the European Union countries (source JRC COVID-19 *In Vitro* Diagnostic Devices and Test Methods Database ^50^). Rapid Antigen Tests (RATs) are recommended to be routinely used, ^51^^,^^52^ especially on oropharyngeal/nasopharyngeal (OP/NP) swab samples. Researchers have had sometimes problems in terms of sensitivity and specificity with some of them. ^53^ Problems may arise because the tests were initially evaluated on samples from patients with severe COVID-19, who are suggested to develop a much higher immune response than those with mild or asymptomatic disease. ^54^

RT-PCR is considered the gold standard method for detection of SARS-CoV-2. However, we had previous experience of multiple negative results SARS-CoV-2 RT-PCR tests on OP/NP swab samples from individuals with a strong clinical suspicion of COVID-19. ^33^ Mardian *et al*. 2021 recommend fecal detection of viral RNA when nasopharyngeal swab data are questionable. ^55^ “In a Systematic Review and Meta-analysis, at the beginning of the pandemic, it was observed that viral RNA was present in the stool in 48.1% of patients during the disease and that 70.3% of patients had prolonged shedding that could extend beyond 33 days from the onset of the disease.” ^56^ Finally, in a recent study aimed to evaluate the role of fecal-oral transmission, unique RNA SARS-CoV-2 genomic sequence mutations have been observed by performing next-generation sequencing on the fecal samples. ^25^ In this case the Luminex technology as molecular testing tool was chosen because it is ideal for fast high-throughput COVID-19 screening and its clinical performance have been evaluated. ^57^

In consideration that SARS-CoV-2 was detected at low levels in fecal samples, ^58^ in addition to molecular test, was agreed to verify the presence of the virus by acquiring images of at least one sample. As proposed by Dittmayer and colleagues, ^22^ in the case of COVID-19 diagnosis, the use of image analysis to confirm the presence of SARS-CoV-2 particles complements the detection of molecular traces of SARS-CoV-2 specific proteins or nucleic acids (and vice versa). Furthermore, as previously demonstrated, we suggest that the ^15^N nitrogen isotope instrument is essential for confirmation of RNA virus replication within bacteria and to observe the bacteriophage behaviour of SARS-CoV-2 in this case. The condition is to obtain faecal matter from the sick patient during the acute phase. ^36^^,^^59^ An integrative study could be carried out by studying the faecal plate and excluding other bacteriophages, as reported in study 36, in which we obtained phage plates on two bacteria, *Faecalibacterium prausnitzii,* and *Dorea formicigenerans*, for which no known bacteriophages were present in the faecal sample culture, using the supernatant derived from the cultures of the SARS-CoV-2 bacteria. In studies of infectious diseases, TEM is used very often to definitively prove the presence of an infectious unit. The images were obtained by TEM, immune-EM, and by fluorescence microscope. What we have noted is (in agreement with our first observations ^30^), that could be present an important role of bacteria in the fecal-oral transmission of SARS-CoV-2. The only limitations of such investigations are the high costs and long waiting times.

### Limits of the present study

In the present study, given the ongoing pandemic crisis during the family study, and considering the intermediate sensitivity and specificity of some nasopharyngeal antigenic tests, we chose to perform molecular tests, with Luminex technology, on faecal samples, given our previous experience described in Ref. 33. However, it should be emphasized that recent authors have greatly improved the genetic search for viral presence. Recent important studies ^60^ have shown how an integrated approach with five commercial RT-PCR kits and a laboratory-developed and validated SYBR-green method, achieves a sensitivity and specificity in nasopharyngeal swabs of over 90%. These studies underline how RT-PCR kits that target many genes have a higher detection rate, resulting in fewer false positives.

## Conclusions

Here we report the case of a child symptomatic for COVID 19, transmitted by one of the parents, whose relatives had tested negative on the rapid antigenic nasopharyngeal swab test. Analyses of fecal samples by high-throughput COVID-19 screening (Luminex technology) allowed us to accurately detect the viral RNA presence in the faces of the child and of all her relatives, which thus resulted to be positive asymptomatic.

Microscopy images analysis was used as complementary approach to confirm the presence of SARS-CoV-2 in bacterial cultures obtained by fecal sample of an infected individual with the viral RNA load positive individual. The images obtained by TEM, immune-EM and by fluorescence microscope show SARS-CoV-2 inside human gut bacteria and outside a matrix resembling extracellular bacterial lysates, in agreement with a bacteriophage mechanism. ^29^ This first observation invites us to pay more attention to the fecal-oral transmission route of the virus and suggests as a further possible reservoir of the virus also the bacteria of the human gut microbiome.

We believe that accurate analysis of the human gut microbiome during viral infections, including SARS-CoV-2 infections, may be of great importance and may aid in diagnosis when other tests fail. ^26^ According to the other studies ^55^ faster and more versatile tests should be improved to decrease or cope with the contagiousness of the pathogens, especially to detect them in the stools. The observation of all clinical symptoms, typically respiratory, gastrointestinal, and neurological, combined with molecular testing (stool, sputum, tear, other fluids) and image analysis, represents the key for understanding the interaction of SARS-CoV-2 with the human gut microbiome and its product. ^61^ Therefore, for the provision of the correct epidemiology, diagnosis and accurate therapeutic approach is important in the treatment of COVID-19, especially in challenging cases, such as the one reported here. This case also highlights the possibility of contagion from asymptomatic parents to their children.

## Materials and Methods notes ^61^^–^^68^

The materials and methods used in this study are deposited at the following links:

https://zenodo.org/records/6974414 ^62^

https://zenodo.org/records/13839323 ^68^

Over the past two years, the various scientific papers and tests in which materials and methods have been described are as follows in references. ^30^^,^^33^^,^^36^^,^^59^^,^^61^^-^^66^

References 36 and 59 present d the methodology on how to use the Nitrogen 15 isotope to be able to determine whether an RNA virus is also bacteriophagic. That work brought to light that Poliovirus also has a bacteriophagic attitude. In manuscript Ref. 36 was resolved and verified all methodological aspects of immunofluorescence and electron microscopy images. We reported only a small text with the permission of the authors: *“Orenstein et al. ^37^ point out, that coronavirus particles can be visible at different densities (particularly ribonucleoprotein). The authors state: “Mature particles had a spherical central core, which was clear, contained electron-dense granules (nucleocapsid) or was totally electron-dense ( … ..)”; … … . Immunofluorescence microscopy, as described by the same authors, was performed according to the manufacturers’ protocol, using primary antibodies against the SARS-CoV-2 nucleocapsid protein “Sars Nucleocapsid Protein Antibody [Rabbit Polyclonal]—500 μg 200-401-A50 Rockland” and the “Goat anti-Rabbit IgG (H+L) Cross-Adsorbed Secondary Antibody, Cyanine3 #A1052” as a secondary antibody. Gram-positive bacteria were stained with a primary antibody (Ab (BDI380), GTX42630 Gene Te”) and “Goat anti-Mouse IgG (H+L), Super-clonal™ Recombinant Secondary Antibody, Alexa Fluor 48” as a secondary antibody. The images confirm the presence of SARS-CoV-2 particles (red light in the fluorescence images) in relationship with the bacteria (green light in the fluorescence images). The control of the specific reactivity of primary antibodies versus Gram-positive bacteria was performed using a culture with a negative molecular test for SARS-CoV-2. It is important to search in the literature if other authors have tested the same antibody and validated it. The control of the specific reactivity of antibodies versus Gram-positive bacteria was assumed from the worl of Kohda et al. and Kameli et al. [……]. In addition, the Gram-positive bacteria antibody [BDI380] Genetex is a mouse monoclonal antibody that has been validated and tested to be reactive toward many Gram-positive bacteria—precisely what we were interested in finding out. The specifications can be found at the following link:* https://www.genetex.com/Product/Detail/Gram-Positive-Bacteria-antibody-BDI380/GTX42630 *(accessed on 20 February 2024). The authors stated the following: “Reactive with lipoteichoic acid (LTA) of many Gram-positive bacteria. Cross-reacts with Listeria monocytogenes (all serotypes), Streptococcus pneumoniae, Staphylococcus aureus, Staphylococcus epidermidis, Enterococcus faecium, Bacillus cereus, Bacillus subtilis and group B Streptococcus (weak). Does not react with Clostridium perfringens”. The control of the specific reactivity of primary antibodies versus the nucleocapsid protein of SARS-CoV-2 was assumed by Zhao et al. […]. In addition, the Rockland primary antibody (Sars Nucleocapsid Protein Antibody [Rabbit Polyclonal]—500 μg 200-401-A50 Rockland) we used for immunofluorescence experiments has no less than 76 references where it has been used, and its remarkable specificity towards SARS-CoV-2 was defined. The references can be seen on the following page:* https://www.rockland.com/catego-ries/primary-antibodies/sars-nucleocapsid-protein-antibody-200-401-A50/#productReferenceSectionWrapper *(accessed on 20 February 2024 … ..”.*

## Consent

Written informed consent for publication of their clinical details and clinical images was obtained from the parents of the child. Written informed consent for publication of their clinical details and clinical images was also obtained from all other patients involved in the study.

## Data availability

All data underlying the results are available as part of the article and are viewable at the following DOI https://doi.org/10.5281/zenodo.6974414, according to the journal guidelines; https://zenodo.org/record/6974414#.YvFp6-xBxmA and https://zenodo.org/records/13839323. ^62^^,^^68^

### Underlying data

All data underlying the results are available as part of the article and no additional source data are required.

### Extended data

Zenodo: Supplementary materials (s.m.) of “The first report on detecting SARS-CoV-2 inside bacteria of the human gut microbiome: A case series on asymptomatic family members and a child with COVID-19” https://doi.org/10.5281/zenodo.6974414. ^62^

This project contains the following extended data:

- Supplementary material: Materials and methods of the tests described in the paper (detection of viral RNA by Luminex method, immunofluorescence at microscopy, electron microscopy, proteomics, and viral protein labeling by nitrogen radioisotope.

Data are available under the terms of the Creative Commons Attribution 4.0 International (CC BY Creative Commons 4.0 license).

## Consent

Written informed consent for publication of their clinical details and clinical images was obtained from the parents of the child. Written informed consent for publication of their clinical details and clinical images was also obtained from all other patients involved in the study.
