# Supplementary material for: The first report on detecting SARS-CoV-2 inside bacteria of  the human gut microbiome: A case series on asymptomatic family members and a child with COVID-19
Source: F1000Res. 2024 Oct 16;11:135. Originally published 2022 Feb 2. [Version 3] doi: 10.12688/f1000research.77421.3 (PMC11502994; doi:10.12688/f1000research.77421.3)
Supplement: Peer review history

# Reviewer report: Reviewer response for version 3

**DOI:** 10.5256/f1000research.172576.r332339

**Published:** 2024-10-24

**Authors:** Debojyoti Dhar

**Affiliations:** 1 Leucine Rich Bio Pvt. Ltd, Bengaluru, Karnataka, India

I hereby approve the manuscript for indexing.

Are enough details provided of any physical examination and diagnostic tests, treatment given and outcomes?

Partly

Is the background of the cases’ history and progression described in sufficient detail?

Partly

Is sufficient discussion included of the importance of the findings and their relevance to future understanding of disease processes, diagnosis or treatment?

No

Is the conclusion balanced and justified on the basis of the findings?

Partly

Reviewer Expertise:

NA

I confirm that I have read this submission and believe that I have an appropriate level of expertise to confirm that it is of an acceptable scientific standard.

# Reviewer report: Reviewer response for version 3

**DOI:** 10.5256/f1000research.172576.r332337

**Published:** 2024-10-17

**Authors:** Hassan Mohammad Al Emran

**Affiliations:** 1 Jashore University of Science and Technology, Jashore, Bangladesh

I have no further comments to make.

Are enough details provided of any physical examination and diagnostic tests, treatment given and outcomes?

Partly

Is the background of the cases’ history and progression described in sufficient detail?

Yes

Is sufficient discussion included of the importance of the findings and their relevance to future understanding of disease processes, diagnosis or treatment?

Partly

Is the conclusion balanced and justified on the basis of the findings?

Yes

Reviewer Expertise:

Virology, infectious diseases

I confirm that I have read this submission and believe that I have an appropriate level of expertise to confirm that it is of an acceptable scientific standard.

# Reviewer report: Reviewer response for version 2

**DOI:** 10.5256/f1000research.136297.r305038

**Published:** 2024-08-21

**Authors:** Hassan Mohammad Al Emran

**Affiliations:** 1 Jashore University of Science and Technology, Jashore, Bangladesh

The article needs two major clarifications. Perhaps additional tests may overcome those weakness.

 1. The RT-PCR test should be performed with nasal/oral samples. Only antigen test is not sufficient to declare as negative.

 2. How it was confirmed that the EM image is of SARS-CoV-2. It could be a bacteriophage. Perhaps you can provide an in vivo cell culture test that will clarify the virus has the capacity to lyse the bacteria.

Are enough details provided of any physical examination and diagnostic tests, treatment given and outcomes?

Partly

Is the background of the cases’ history and progression described in sufficient detail?

Yes

Is sufficient discussion included of the importance of the findings and their relevance to future understanding of disease processes, diagnosis or treatment?

Partly

Is the conclusion balanced and justified on the basis of the findings?

Yes

Reviewer Expertise:

Virology, infectious diseases

I confirm that I have read this submission and believe that I have an appropriate level of expertise to confirm that it is of an acceptable scientific standard, however I have significant reservations, as outlined above.

## References

[1] : Evaluation of RT-PCR assays for detection of SARS-CoV-2 variants of concern. *Sci Rep* .2023;13(1) : 10.1038/s41598-023-28275-y 2342 10.1038/s41598-023-28275-y 36759632 PMC9910272

# Response

**Published:** 2024-09-26

**Authors:** Carlo Brogna

**Affiliations:** Craniomed group facility, Italy

In bold our answers

 R :

1. We fully agree with the reviewer. Unfortunately, considering that we were in an emergency and that the reliability of antigenic tests was unclear, we preferred to perform molecular tests at Luminex on faecal samples in this case. We have included the reasons and the need for molecular test, as suggested by the reviewer, in the last paragraph of the discussion:

« Limits of the present study:

 In the present study, given the ongoing pandemic crisis during the family study, and considering the intermediate sensitivity and specificity of some nasopharyngeal antigenic tests, we chose to perform molecular tests, with Luminex technology, on faecal samples, given our previous experience described  in 33.  However, it should be emphasized that recent authors have greatly improved the genetic search for viral presence. Recent important studies (60) have shown how an integrated approach with five commercial RT-PCR kits and a laboratory-developed and validated SYBR-green method , achieves  a sensitivity and specificity in nasopharyngeal swabs of over 90%. These studies underline how RT-PCR kits that target many genes have a higher detection rate, resulting in fewer false positives. »

 2. We thank the reviewer for suggesting how to improve this work, and in agreement with him, we have indicated the studies in which we performed the 15N nitrogen isotope experiment in bacterial cultures in which SARS-CoV-2 was present and the detection of nitrogen in virus proteins. We have also included and detailed the experiment reproduced in our other work on phage plates. In the present work we have written thus:

*« In addition we suggest, as previously demonstrated that the tool of Nitrogen isotope 15N is essential to have confirmation of the replication of the RNA virus inside the bacteria and observe the bacteriophage behavior of SARS-CoV-2 in this case. The condition will be to obtain the fecal matter from  sick patient during the acute phase( 36, 59). An integrative study could be performed with the study of the lytic plaque and the exclusion of other bacteriophages as reported in the study 36, where we have obtained phage plates on two bacteria, Faecalibacterium prausnitzii and Dorea formicigenerans, for which no known bacteriophages were present in the fecal sample culture, using the supernatant derived from the SARS-CoV-2 bacteria cultures »*

 References  as reported in the major manuscript:

 33. Brogna B, Brogna C, Petrillo M, et al.: SARS-CoV-2 Detection in Fecal Sample from a Patient with Typical Findings of COVID-19 Pneumonia on CT but Negative to Multiple SARS-CoV-2 RT-PCR Tests on Oropharyngeal and Nasopharyngeal Swab Samples. Medicina (Kaunas). 2021 Mar 20;57(3):290. 33804646 10.3390/medicina57030290 PMC8003654

 36. Brogna C, Bisaccia DR, Costanzo V, et al. Who Is the Intermediate Host of RNA Viruses? A Study Focusing on SARS-CoV-2 and Poliovirus. Microorganisms. 2024;12(4):643. Published 2024 Mar 23. doi:10.3390/microorganisms12040643

 59.  Brogna C, Cristoni S. A new absolute quantitative method for peptide and metabolite detection. J Mass Spectrom. 2024 Jan;59(1):e4991. doi: 10.1002/jms.4991. PMID: 38108532.

 60. Dip SD, Sarkar SL, Setu MAA, Das PK, Pramanik MHA, Alam ASMRU, Al-Emran HM, Hossain MA, Jahid IK. Evaluation of RT-PCR assays for detection of SARS-CoV-2 variants of concern. Sci Rep. 2023 Feb 9;13(1):2342. doi: 10.1038/s41598-023-28275-y. PMID: 36759632; PMCID: PMC9910272.

 We thank the reviewer for helping to improve this work

# Reviewer report: Reviewer response for version 2

**DOI:** 10.5256/f1000research.136297.r146819

**Published:** 2023-04-05

**Authors:** Aravind Natarajan

**Affiliations:** 1 Department of Genetics, Stanford University, Stanford, CA, USA

The revised manuscript from Brogna *et al* addresses some of the concerns raised by the reviewers. It now includes a) a supplementary file with some methodological details, b) editorial changes and additional references for clarity, and c) controls in the fluorescence microscopy data in figure 3. Revisions to the manuscript and detailed responses to comments from reviewers warrant an additional review of the manuscript. This review is written in parts, independently addressing the conclusions listed in the current study.

**Conclusion 1:** SARS-CoV-2 RNA is shed in stool by asymptomatic patients. The viral RNA can be detected for an extended period of time, up to 90 days. Gastrointestinal (GI) symptoms are an important manifestation of COVID-19 infection. These observations suggest that patients suspected of COVID-19 should be subject to stool tests in addition to testing of respiratory samples, and GI symptoms should be considered in their diagnosis. Further, public health measures should be wary of potential oro-fecal transmission of SARS-CoV-2. 

**Relevant data:** The manuscript outlines the case of a 21-month-old female Caucasian child who manifested bilateral conjunctivitis, lacrimation, diarrhea, malodorous stools, restlessness, and fever, leading to an evaluation of all of her family members. While the child and all of her family members tested negative on a rapid antigen test performed on nasopharyngeal swabs, the current study found their stool to be positive for SARS-CoV-2-RNA detected using an RT-PCR-based assay on the Luminex platform. Finally, this study finds that one adult male member of the family had prolonged presence of SARS-CoV-2 RNA in stool until 90 days after initial diagnosis.

**Comments:**

**a.** This manuscript provides unambiguous evidence to support conclusion 1 summarized above. This result is in keeping with others’ findings in the literature. 

**b. **Bilateral conjunctivitis and lacrimation are not known to be associated with COVID-19. The authors provide a specious explanation that these symptoms may be mediated by the effect of the infection on the gut microbiota. While there is preliminary data that a COVID-19 infection can affect the gut microbial composition, that such changes could lead to bilateral conjunctivitis or lacrimation are unfound.

**Conclusion 2:** SARS-CoV-2 replicates in bacterial cells from the gut. The authors strive to prove this through three orthogonal methods - i) *in vitro* culturing, ii) transmission electron microscopy and iii) fluorescence microscopy.

**i) *In vitro culturing***

**Relevant data:** Stool was collected from an asymptomatic adult male and tested to contain SARS-CoV-2 RNA using the Luminex assay. Further, the sample was homogenized, washed with 50 mM NaHCO3, resuspended in 10 mM NaCl, and inoculated in NutriSelect Plus nutrient broth. The culture was grown aerobically at 37°C for 30 days. In these 30 days, the authors report an increase in viral RNA concentration from 24 to 520 arbitrary units.

**Comments:**

**a.** Methodology associated with this experiment lacks clarity. How long were the stool samples stored at 4°C before being processed? The duration of storage prior to processing can influence the microbial (bacterial and virus) constituents of stool samples. Why was the stool sample diluted in water as opposed to a buffer like phosphate-buffered saline (PBS) that avoids osmotic stress and better recovery of microbes? How much of the homogenized stool sample was washed in 50 mM NaHCO3? Why was the homogenized stool sample washed with 50 mM NaHCO3? Why was the final washed pellet from the stool sample resuspended in 10 mM NaCl? How much of this resuspended sample was added to how much nutrient broth in a tube or flask of what size? Why was the culture grown in aerobic conditions while many bacteria in the gut are anaerobic? The methods section in supplementary materials suggests that the culture was grown for one week while the main manuscript suggests that it was grown for 30 days - which one is it?  Without these details, the experiment carried out in this work can not be replicated and the results presented cannot be fully understood. Choices made in deciding how to process the stool sample and culture microbes greatly influence what microbes are favored to grow. Given that the authors investigate whether SARS-CoV-2 replicates in certain gut bacteria, these choices regarding culture conditions are important variables.

**b. **The viral RNA concentration is reported in arbitrary units, leading to a lack of clarity. One of the crucial pieces of evidence in favor of the hypothesis that viruses replicate in the *in vitro* setup herein is the increase in detected viral RNA over time. This data needs to be reported in absolute units and not arbitrary units, through the use of standards. This is because it is unclear what mathematical relationship the arbitrary units share with the concentration of RNA. Table 1s in supplementary materials is unreadable given missing information on the identity of the rows and missing units in the columns. Further, the current study also does not clarify what the limit of detection of their assay is. These comments from the previous review are yet to be addressed.

**c. **The methods section in the supplementary material mentions monitoring bacterial growth by measuring optical density. This data is missing in the manuscript.

**d.** How did the study account for the evaporation of media over an extended period (one week/ 30 days) of culturing at 37°C?

**e. **If the authors believe that the SARS-CoV-2 virus capable of infecting humans can replicate in bacterial cultures from stool samples, then, the authors were effectively growing contagious SARS-CoV-2 virus in the lab. Given that their experiment involved incubating replicating viral cultures in an orbital shaker where microbes are aerosolized, what precautions did the authors take to prevent transmission of the virus? This is important information for future replication of experiments herein.

 While the increase in viral RNA concentration over time is an interesting preliminary observation suggesting replication of viral RNA *in vitro*, the experiments herein can neither be appropriately reviewed nor replicated given the lack of methodological information and data. Finally, stool samples are a highly complex matrix with numerous variables. If the hypothesis that bacteria in the gut can foster the replication of the SARS-CoV-2 virus *in vitro* is true, have the authors attempted to grow purified stocks of SARS-CoV-2 virus in bacterial cultures derived from stool samples from healthy participants?

**ii) Transmission electron microscopy**

**Relevant data:** Bacterial cultures derived from SARS-CoV-2 +ve stool, grown *in vitro* in NutriSelect Plus nutrient broth for 30 days were pelleted and processed for TEM. Sections of the fixed sample were stained with primary anti-SARS-CoV-2 nucleocapsid protein antibody and secondary anti-rabbit gold-conjugated antibody. Authors point to features of the TEM image pre and post-embedding as SARS-CoV-2 particles in figure 2. They include bacterial culture derived from the stool of a healthy patient and not stained with the primary antibody as a negative control.

**Comments:** The TEM data is crucial to determining whether or not SARS-CoV-2 viral particles are indeed found inside bacterial cells. However, the lack of ultrastructural features typical of SARS-CoV-2 in figures 2A and B makes this data inconclusive. Notably, in figure 2A the manuscript points to a vesicle containing smaller vesicles as an example of SARS-CoV-2 within a bacterial cell. However, the host bacterial cell displays no cellular features typical of bacterial cells. Finally, the manuscript lacks appropriate controls for data in figure 2C. For instance, figure 2D, which is meant to be one of the controls required, does not include the primary antibody, making this an insufficient control. Finally, the robustness of antibody based labeling of samples is highly dependent on experimental conditions. Therefore controls are crucial and immunogenicity of antibodies cannot be assumed from previous studies, as cited in the supplementary information.

 Given these shortcomings, it is not clear that the TEM images in fact capture SARS-CoV-2 viruses within bacterial cells.

**iii) Fluorescence microscopy**

**Relevant data:** Supernatant from bacterial cultures derived from SARS-CoV-2 +ve stool grown *in vitro* in NutriSelect Plus nutrient broth for 30 days was processed for immunofluorescence. Slices of samples fixed in PFA were probed with primary antibodies against the viral nucleocapsid protein and gram-positive bacteria. Secondary antibodies with orthogonal fluorescent labels enable simultaneous detection of the SARS-CoV-2 nucleocapsid protein as red fluorescence and gram-positive bacteria as green fluorescence.

**Comments:**

**a.** Why were aliquots of the supernatant of the culture used when in fact we want to visualize the bacterial constituents? How was the culture spun down to yield this supernatant?

**b.** The robustness of antibody based labeling of samples is highly dependent on experimental conditions. Therefore controls are crucial and immunogenicity of antibodies cannot be assumed from previous studies, as cited in the supplementary information. The controls required for this experiment are as follows:

- Does the primary antibody against the nucleocapsid protein detect the nucleocapsid protein?
- This is appropriately established in figures 1s A-D.
- Does the primary antibody against the nucleocapsid protein detect anything else in the bacterial cultures derived from the patient stool sample that is not the SARS-CoV-2 nucleocapsid protein?
- This is difficult to establish because stool is a complex sample with a lot of variation across individuals. Therefore, bacterial cultures derived from stool samples acquired from two different individuals are not comparable. The nearest comparison, though imperfect, would be a bacterial culture derived from the same patient after his stool sample turned negative for SARS-CoV-2 RNA at the 90-day mark. The manuscript includes a more imperfect control sample derived from a healthy 18-month-old child who has not been infected by SARS-CoV-2. The methods do not clarify how this healthy stool sample was processed and how samples towards microscopy were prepared. Finally, the methods and figure caption suggest that these samples were probed with both the relevant primary antibodies but make no mention of whether secondary antibodies were included.
- Does the primary antibody against gram-positive bacteria detect gram-positive bacteria?
- This can be established by probing cultures of various gram-positive bacteria with the said antibody.
- Does the primary antibody against gram-positive bacteria detect anything else in the bacterial cultures derived from the patient stool sample that is not gram-positive bacteria?
- This can be established by treating the bacterial cultures with gram-positive specific antibiotics such as vancomycin, leading to cultures that are bereft of gram-positive bacteria. Samples from these cultures when probed with the primary antibody against gram-positive bacteria should not provide any signal.

**c.** In figure 3A-E. the morphology of the bacteria indicated by the yellow boxes as carrying the SARS-CoV-2 virus is rather varied. Can the authors comment on what their interpretation of this result maybe?

 Given shortcomings in methodology and the absence of appropriate controls, it is not definitive that bacteria in fact co-localize with SARS-CoV-2 viral particles.

**iv) Supplementary data**

**Comment on validation of antibody against nucleocapsid protein in figures 1s A-D**

 This establishes that the primary antibody against the SARS-CoV-2 nucleocapsid protein does in fact bind the SARS-CoV-2 nucleocapsid protein and is an important control.

**Comment on the increase in the intensity of fluorescence**

 In the absence of any methodological details, I am unable to comment on this data.

**Comment on nitrogen isotope assay**

 Methodological details are unclear. What component of the medium is labeled with 15N? How was 0.20 g of the labeled medium added in an aseptic manner? After 7 days, how was the culture processed to enrich for proteins? What equipment(s) was used for mass spec? Were there other labeled peptides that are not from SARS-CoV-2 (such as native bacterial peptides) that appeared in the assay?

 At the outset, it is noteworthy that SARS-CoV-2 related proteins bearing labeled 15N were obtained from this experiment. This does provide evidence that SARS-CoV-2 proteins are being expressed in the culture. The conclusion that this points to bacteria replicating viral RNA and viral proteins is specious.

 Did the authors isolate 15N labeled viral RNA? This could be evidence of replication of viral RNA in the culture.

**Comment on conversion in arbitrary units**

 Table 1S is unreadable because the identities of the rows are unstated and the units of the columns are missing.

**Comment on plaque assays**

 It is well known that stool samples are brimming with bacteriophages. It is not at all surprising that medium inoculated with supernatant from stool samples would in fact contain bacteriophages that form plaques. Did the authors try this same assay using the stool sample derived from the same patient at the 90-day time point when their stool was SARS-CoV-2 negative?

**Summary:** The current study, in keeping with numerous previous studies, clearly highlights the importance of testing stool samples for SARS-CoV-2 RNA even in asymptomatic patients suspected of COVID-19 infection. It also notes the importance of paying attention to gastrointestinal symptoms in the diagnosis of COVID-19 infections.

 However, there is insufficient evidence regarding whether SAR-CoV-2 is detected inside bacteria from the human gut, and claims of bacteria hosting SARS-CoV-2 replication are specious at best. Therefore, I recommend that the current manuscript not be approved for indexing. Further, I recommend that the editorial team retract this article.

 Finally, there is a striking absence of key methodological details and controls across the manuscript. This makes a careful review of the results presented herein challenging and replication of experiments impossible. Therefore, I recommend that the current manuscript not be indexed. Further, I recommend that the editorial team retract this article.

Are enough details provided of any physical examination and diagnostic tests, treatment given and outcomes?

Yes

Is the background of the cases’ history and progression described in sufficient detail?

Yes

Is sufficient discussion included of the importance of the findings and their relevance to future understanding of disease processes, diagnosis or treatment?

Yes

Is the conclusion balanced and justified on the basis of the findings?

No

Reviewer Expertise:

Fecal shedding of SARS-CoV-2 RNA

I confirm that I have read this submission and believe that I have an appropriate level of expertise to state that I do not consider it to be of an acceptable scientific standard, for reasons outlined above.

# Reviewer report: Reviewer response for version 1

**DOI:** 10.5256/f1000research.81407.r138763

**Published:** 2022-07-01

**Authors:** Aravind Natarajan

**Affiliations:** 1 Department of Genetics, Stanford University, Stanford, CA, USA

**Brogna C *et al.* The first report on detecting SARS-CoV-2 inside human fecal-oral bacteria: A case series on asymptomatic family members and a child with COVID-19**

 This manuscript by Brogna C *et al.* reports the first observation of SARS-CoV-2 “inside human fecal-oral bacteria” suggesting that SARS-CoV-2 can infect bacteria like a bacteriophage. This study is inspired by a symptomatic 21-month-old child whose nasopharyngeal sample tested negative on a rapid antigen test, but feces tested positive for SARS-CoV-2 viral RNA on a Luminex assay. This led the authors to carry out a contact tracing study, testing stool samples from family members that lived in the same building as the child for SARS-CoV-2 RNA; they found all the family members to be asymptomatic and positive for viral RNA in their stool. Notably, the child’s male parent (am1) continued to have extended shedding of viral RNA out to 90 days from the start of treatment. The authors collected stool samples from this parent to understand the pathobiology of SARS-CoV-2.

 Specifically, they expand on their previous observation (featuring two shared authors with the current manuscript) that this virus could potentially display bacteriophage-like behavior and infect bacteria. In this previous work, they carried out *in vitro* culturing assays and reported that the concentration of viral RNA increased with time when incubated with fecal bacteria ^1^. In another publication (featuring seven shared authors with the current manuscript), the authors use TEM and immunofluorescence microscopy and report visualizing SARS-CoV-2 particles in gut bacteria ^2^. In the current work, they feature these same *in vitro* and microscopy experiments with a different stool sample and report the same conclusion - that bacteria in the gut are infected with SARS-CoV-2 viral particles.

 I recommend this manuscript be rejected in its current form. It may be sent out for reviews again pending major revisions.

**Major suggestions:**

1. Throughout the manuscript, the authors suggest the fecal-oral transmission of SARS-CoV-2. However, whether there are infectious viral particles in stool capable of being transmitted is at best still debated, with more evidence to the contrary ^3^. Especially given the relevance of this matter to clinical decisions and public health, I encourage the authors to present a balanced view.
2. The authors report viral RNA concentration determined through Luminex in arbitrary units (AU). However, my understanding is that Luminex can be set up to contain standards that reveal an absolute concentration of viral RNA. Given how viral RNA concentrations are central data to the conclusions in this work, I believe that reporting the absolute concentration of viral RNA is important. Additionally, this will make the observations here more replicable across labs and viral RNA detection techniques.
3. Regarding the reported viral RNA concentrations, it is unclear what the specificity, sensitivity, and detection range of the Luminex assay are as carried out by the authors. Therefore, I recommend the authors include relevant controls to estimate these. Reporting this information along with their experiments will add important validity and context to the data.
4. Fig 3 is missing key controls. Recommend including a control of uninfected bacteria from a healthy donor stained with both α-SARS-CoV-2 nucleocapsid protein antibody and α-Gram-positive bacteria antibody.
5. The authors conclude from the microscopy data that fecal bacteria are infected by SARS-CoV-2 in samples collected on day 30 from an adult patient, am1. Notably, this patient along with the others in the study was prescribed probiotics with two Gram-positive bacteria ( *Lactobacillus reuteri* and *Bacillus clausii*) in their diets for 30 days prior to sample collection. In the event that bacteria are in fact infected by SARS-CoV-2, it is unclear if this is an artefact of the probiotic supplements and treatment regime. 
6. I encourage the authors to provide more methodological information in the manuscript. As a matter of principle, manuscripts should in and of themselves provide sufficient information for readers to repeat experiments without having to go down the rabbit hole of chasing down references to other works. The current manuscript exhibits this concern that is prevalent in scientific publications. Some instances of this in the current manuscript include - 
  - “molecular testing for SARS-CoV-2 was performed by using Luminex technology as described by us previously” - It is unclear how the samples were prepared, if controls were included and what the parameters of this assay were.
  - Methodology used to isolate and culture bacteria from stool - It is unclear how bacteria were isolated from stool. This is important information to get a sense of how rigorous this method was, whether there are inherent biases regarding what bacteria are favored in the culturing process and if there are chances for other residual contaminants from the fecal sample.
  - Methodology used to collect and preserve stool samples - the majority of existing reports are unable to culture viable SARS-CoV-2 from fecal samples, even when the samples are preserved in Viral Transport Media (VTM). Therefore, clarity about how the current work collected and preserved samples are crucial to understanding how they have been successful at recovering viable viral particles.

 

**Minor suggestions:**

1. Request citation and clarity on the statement - “several studies have reported….intestinal bacterial co-infection in COVID-19 patients”.
2. Request citation and clarity on the statement - “Observations of possible links between the animal gut microbial environment and coronaviruses have been reported over time”. The current citations do not have evidence for a possible link to the gut microbial environment.
3. Fig 1 reports “Viral load” although what is actually reported is the viral RNA concentration, which can be different from viral load because not all viral particles are lysed and provide RNA for detection, and some viral RNA is reported to come from non-viral reservoirs ^4^.
4. The manuscript calls for major editorial revisions given numerous improper usages of phrases. For instance - a) There are no “fecal-oral bacteria”, which suggests specific bacteria in the feces are able to be transmitted orally. b) It is unclear what “Bacterial feces” means. c) “RNA viral load count” appears miswritten.

**Additional notes:**

1. I don’t have experience with interpreting TEM images and therefore leave this comment here rather than as a cause for major revision. TEM images are complex requiring the appropriate collection and processing of samples, the inclusion of controls, and careful interpretation of observations. The improper use of EM to study SARS-CoV-2 from tissue samples has been a cause for concern ^5^^,^^6^. To my untrained eye, Fig 2 seems to be another example of misinterpreted EM data because - 
  - If Fig 2a in fact displays SARS-CoV-2 viral particles inside a bacterium, it is surprising to me that the bacterial host appears to be entirely filled by viral particles with no room for its essential, native molecules. To me, this figure seems like that of the widely described multivesicular bodies that are unrelated to SARS-CoV-2 and yet regularly misinterpreted ^6^.  
  - The figures don’t appear to have sufficient resolution to highlight the ultrastructures typical of SARS-CoV-2 that are required to unmistakable identify viral particles ^5^.
  - If Fig 2b in fact presents the case of a bacteria with viral particles around it, it merely appears like viral particles proximal to the bacteria in this image, with no evidence that these viruses in fact infected the bacteria. The conclusion that viral particles are found in the extracellular matrix of gut bacteria is an over-reach without sufficient evidence.
  - The figure is missing controls such as stained and unstained images of comparable bacteria without the presence of the alleged SARS-CoV-2 viral particles. Given these observations and the importance of this figure to the central tenet of this manuscript, I recommend the editorial team seeks input from an expert in TEM.
2. The hypothesis that SARS-CoV-2 may infect bacteria is both biologically fascinating and clinically highly relevant. This is because there are no examples that I know of or can find of viruses that affect humans that can also retain the molecular machinery to affect bacterial cells. Therefore, the idea that this is possible is biologically fascinating and can pave the path to many follow-up studies. Further, SARS-CoV-2 continues to be a major threat, having claimed over 6 million people worldwide. Therefore, careful evaluation of the potential sources of future outbreaks is critical from a personal and public health standpoint. If in fact bacteria in the gut can harbor infectious SARS-CoV-2 viruses, this adds to another dimension of the COVID-19 pandemic that calls for urgent precautionary measures. Unfortunately, the current manuscript is missing many controls and methodological details, and has insufficient data to make the case that bacteria are in fact infected by SARS-CoV-2. Given the hugely significant impact that concluding gut bacteria can be infected by SARS-CoV-2 through a bacteriophage-like property can have to humanity, I encourage the authors and editorial team to exercise caution and responsibility in promoting this conclusion with insufficient evidence.

Are enough details provided of any physical examination and diagnostic tests, treatment given and outcomes?

Yes

Is the background of the cases’ history and progression described in sufficient detail?

Yes

Is sufficient discussion included of the importance of the findings and their relevance to future understanding of disease processes, diagnosis or treatment?

Yes

Is the conclusion balanced and justified on the basis of the findings?

No

Reviewer Expertise:

Fecal shedding of SARS-CoV-2 RNA

I confirm that I have read this submission and believe that I have an appropriate level of expertise to state that I do not consider it to be of an acceptable scientific standard, for reasons outlined above.

## References

[1] : Increase of SARS-CoV-2 RNA load in faecal samples prompts for rethinking of SARS-CoV-2 biology and COVID-19 epidemiology. *F1000Res* .2021;10: 10.12688/f1000research.52540.3 370 10.12688/f1000research.52540.3 34336189 PMC8283343

[2] : Could SARS-CoV-2 Have Bacteriophage Behavior or Induce the Activity of Other Bacteriophages?. *Vaccines (Basel)* .2022;10(5) : 10.3390/vaccines10050708 10.3390/vaccines10050708 35632464 PMC9143435

[3] : Rectally shed SARS-CoV-2 lacks infectivity: time to rethink faecal–oral transmission?. *Nature Reviews Gastroenterology & Hepatology* .2021;18(9) : 10.1038/s41575-021-00501-w 10.1038/s41575-021-00501-w PMC831163234312524

[4] : SARS-CoV-2 genomic and subgenomic RNAs in diagnostic samples are not an indicator of active replication. *Nature Communications* .2020;11(1) : 10.1038/s41467-020-19883-7 10.1038/s41467-020-19883-7 PMC769571533247099

[5] : Why misinterpretation of electron micrographs in SARS-CoV-2-infected tissue goes viral. *The Lancet* .2020;396(10260) : 10.1016/S0140-6736(20)32079-1 e64-e65 10.1016/S0140-6736(20)32079-1 PMC753580233031763

[6] : Multivesicular bodies mimicking SARS-CoV-2 in patients without COVID-19. *Kidney International* .2020;98(1) : 10.1016/j.kint.2020.05.003 233-234 10.1016/j.kint.2020.05.003 32437766 PMC7206432

# Response

**Published:** 2022-07-17

**Authors:** Carlo Brogna

**Affiliations:** Craniomed group facility, Italy

**Reviewer comments** **and Brogna et al. responses (R.)**

 Brogna C et al. The first report on detecting SARS-CoV-2 inside human fecal-oral bacteria: A case series on asymptomatic family members and a child with COVID-19

 This manuscript by Brogna C et al. reports the first observation of SARS-CoV-2 “inside human fecal-oral bacteria” suggesting that SARS-CoV-2 can infect bacteria like a bacteriophage. This study is inspired by a symptomatic 21-month-old child whose nasopharyngeal sample tested negative on a rapid antigen test, but feces tested positive for SARS-CoV-2 viral RNA on a Luminex assay. This led the authors to carry out a contact tracing study, testing stool samples from family members that lived in the same building as the child for SARS-CoV-2 RNA; they found all the family members to be asymptomatic and positive for viral RNA in their stool. Notably, the child’s male parent (am1) continued to have extended shedding of viral RNA out to 90 days from the start of treatment. The authors collected stool samples from this parent to understand the pathobiology of SARS-CoV-2.

 Specifically, they expand on their previous observation (featuring two shared authors with the current manuscript) that this virus could potentially display bacteriophage-like behavior and infect bacteria. In this previous work, they carried out in vitro culturing assays and reported that the concentration of viral RNA increased with time when incubated with fecal bacteria1. In another publication (featuring seven shared authors with the current manuscript), the authors use TEM and immunofluorescence microscopy and report visualizing SARS-CoV-2 particles in gut bacteria2. In the current work, they feature these same in vitro and microscopy experiments with a different stool sample and report the same conclusion - that bacteria in the gut are infected with SARS-CoV-2 viral particles.

 I recommend this manuscript be rejected in its current form. It may be sent out for reviews again pending major revisions.

**Major suggestions:**

 Throughout the manuscript, the authors suggest the fecal-oral transmission of SARS-CoV-2. However, whether there are infectious viral particles in stool capable of being transmitted is at best still debated, with more evidence to the contrary3. Especially given the relevance of this matter to clinical decisions and public health, I encourage the authors to present a balanced view.

**R: First, we are honored by the reviewer's comments on the fecal-oral route of transmission od the virus SARS-CoV-2 because they were our doubts at the beginning of the pandemic and were the reason we wanted to do the check on prokaryotic cells. In fact, we found it really embarrassing that no major research group in the world has documented a positive or also negative control on the interaction between Coronavirus, in general in the last 30 years, and SARS-CoV-2 in the last 2 years and prokaryotic unicellular cells.**

**Only the famous diatribes on the occurrence of bacterial cofactor in HIV (RNA virus) infection can be found in the literature.  We respect the reviewer's opinion on the epidemiology and transmission route of the virus, but we believe that in a balanced context, the oro-fecal route of transmission should also, and not only be considered. Reference 3 that the reviewer cites is a letter,  a commentary by Pedersen et al. to the work of Guo et al. (7), a manuscript that collected a review of other works emphasizing the fecal-oral transmission pathway. Guo et al. (8) responded to the letter from Pedersen et al., including more recent work and reiterating that the oro-fecal route of transmission must be considered. In addition, it should be considered that historically the coronaviruses family, which afflict animals in general, have long been listed in the literature as viruses with an oro-fecal route of transmission (many studies are present in the literature). Our third paper (2) supported the addition of the oro-fecal transmission route, including many new studies in this regard after the publication of the commentary by Pedersen et al. Since the beginning of the pandemic, several authors have pointed out that SARS-CoV-2 has a close relative: the coronavirus RATG13 (9). RATG13 was discovered in 2013, and picked up by researchers following the deaths of 3 out of 6 miners working in caves in Mojiang county in Yunnan, China (10), as currently recognized. The miners worked in caves where bat guano (stool) was present, and it is really hard to imagine that in contact with such material, the transmission route was respiratory.**

**However, the reviewer pays attention to this issue, and in line with his opinion, we have modified the text by adding the word " *also*" or " *possible*" next to "transmission route" where this was possible.**

 The authors report viral RNA concentration determined through Luminex in arbitrary units (AU). However, my understanding is that Luminex can be set up to contain standards that reveal an absolute concentration of viral RNA. Given how viral RNA concentrations are central data to the conclusions in this work, I believe that reporting the absolute concentration of viral RNA is important. Additionally, this will make the observations here more replicable across labs and viral RNA detection techniques.

**R: We thank the reviewer for this observation and have included the conversion table with the corresponding formula in the supplementary materials (s.m.), table 1s of paragraph 4, in the second section, "Extra sample processing and extra data. »**

***The total turn around time was around 4 h. Luminex detection was reported in arbitrary units given in accordance with Florida et al.* (4 in s.m. and 11 here) (Line 63-64 s.m. and 265-269 s.m.)**

 Regarding the reported viral RNA concentrations, it is unclear what the specificity, sensitivity, and detection range of the Luminex assay are as carried out by the authors. Therefore, I recommend the authors include relevant controls to estimate these. Reporting this information along with their experiments will add important validity and context to the data.

**R: More details have been included in the supplementary materials (s.m.), paragraph 6 of the first part of Materials and Methods: " *Luminex technology (Life Technology, USA) (ref. 3 in s.m. and 12 here,) was used to detect the viral RNA load in bacterial cultures. The detection was performed by using NxTAG® CoV Extended Panel, a real-time reverse transcriptase PCR assay detecting three SARS-CoV-2 genes on the NxTAG-enabled System MAGPIX® instrument, and the AccuPlex™ SARS-CoV-2 Reference Material Kit (SeraCare) as reference standard with sequences from the SARS-CoV-2 genome."***

 Fig 3 is missing key controls. Recommend including a control of uninfected bacteria from a healthy donor stained with both α-SARS-CoV-2 nucleocapsid protein antibody and α-Gram-positive.bacteria antibody.

**R: We agree with the reviewer that controls, in general, are essential. That's why it seemed, first of all, absurd to us that no researcher had designed tests to check for interactions between coronaviruses and prokaryotic cells. This is why we emphasized that it is important to do different tests with different methods so that each is a control of the other, and that is what we did in Brogna et. al (2) and repeat in the supplementary materials, now added. For the required control with fluorescence vision of gram+ bacteria and lack of fluorescence for viral proteins in question, a sample of stool from an healthy 18-month-old child, checked negative by  molecular testing,  with healthy parents and never ill with SARS-CoV-2 at the time of collection, was used. This choice, a child's faeces, was made because, after two years of the pandemic, it is very difficult to find uninfected or asymptomatic carriers. However, as usual, we had not put in the panels in Figure 3 such control though we had done so. We thought the evidence in panel E of figure 3 would be sufficient. However, the reviewer's suggestion is valuable, and we add the required control (figure 3 panels F and G)and comment on it below:**

**A panel (F, G) was added with the required control, a culture of only bacteria from the feces of a healthy individual, an 18-month-old child to be exact. The sample was obtained with the consent of both parents.  As can be seen, only gram+ bacteria are highlighted by fluorescence, although both primary antibodies have been included, both to gram+ and to the nucleocapsid protein of SARS-CoV-2.  The sentence "... *and panels F and G show a group of gram+ bacteria by fluorescence, derived from the stool bacteria culture of a healthy 18-month-old child (* *with healthy parents and never ill with SARS-CoV-2 at the time of collection and with* *and with their written consent*) *negative to molecular test to SARS-CoV-2, although the other primary antibody to the nucleocapsid protein is also included and does not show a red signal*". Line 193-199**

 The authors conclude from the microscopy data that fecal bacteria are infected by SARS-CoV-2 in samples collected on day 30 from an adult patient, am1. Notably, this patient, along with the others in the study was prescribed probiotics with two Gram-positive bacteria (Lactobacillus reuteri and Bacillus clausii) in their diets for 30 days prior to sample collection. In the event that bacteria are in fact infected by SARS-CoV-2, it is unclear if this is an artefact of the probiotic supplements and treatment regime. 

**R: We apologize to the reviewer if we are not clear. The Luminex data at day 60 refer to subsequent stool sampling from family members, whereas the microscope images are from bacteria culture of the am1 sample taken at time zero and cultured, as detailed in the supplementary materials, for 30 days in the laboratory.**

**In addition, the probiotic legend was included because it is required by the case series checklist guidelines but should not be understood as an indication of possible therapy. The use of probiotics was used only as an aid in view of the fact that all the literature now agrees that SARS-CoV-2-induced alteration of bacterial flora occurs. The words « *only as re-balancers of bacterial flora*… » are added. Line101 e 127**

 I encourage the authors to provide more methodological information in the manuscript. As a matter of principle, manuscripts should in and of themselves provide sufficient information for readers to repeat experiments without having to go down the rabbit hole of chasing down references to other works. The current manuscript exhibits this concern that is prevalent in scientific publications. Some instances of this in the current manuscript include - 

 “molecular testing for SARS-CoV-2 was performed by using Luminex technology as described by us previously” - It is unclear how the samples were prepared, if controls were included and what the parameters of this assay were.

 Methodology used to isolate and culture bacteria from stool - It is unclear how bacteria were isolated from stool. This is important information to get a sense of how rigorous this method was, whether there are inherent biases regarding what bacteria are favored in the culturing process and if there are chances for other residual contaminants from the fecal sample.

 Methodology used to collect and preserve stool samples - the majority of existing reports are unable to culture viable SARS-CoV-2 from fecal samples, even when the samples are preserved in Viral Transport Media (VTM). Therefore, clarity about how the current work collected and preserved samples are crucial to understanding how they have been successful at recovering viable viral particles.

**R: We thank the reviewer for this valuable advice and apologize because it was not our intention to direct him to read our other work, but unfortunately, as the manuscript is set up as a case series, we had a predetermined number of words and checklist guidelines to follow. To overcome this problem, we suggested that he read our other papers, but the reviewer rightly asked for more details, and we have summarized the materials and methods of our other studies in the supplementary materials (s.m.) now attached. Of course, the words are in italics because we are reporting exactly what we have already published, with author’s permission.**

**Please forgive the reviewer if we take the liberty to give some advice, since this is an open review, to anyone who would like to start repeating or improving the published experiments.  Virus (SARS-CoV-2)-positive stool samples can be stored at 4°C in a previously sterile container as soon as they are collected from the patient. Culture in a multipotent medium, performed to grow only bacterial cells and exclude other eukaryotic cells, started after the homogenization and inorganic debris removal procedures, is best performed within 24 to 48 hours. Therefore, good timing between clinical collection and processing in the appropriate laboratory is important.  In addition, freezing the samples, although it results in the preservation of proteins and nucleic acids, ruins the viability of bacteria.**

**Variations to the presented methodology can be made, or protein expression can be assessed by other methods and quantified over time. Our humble opinion suggests that the genetic part should always be verified with the actual protein expression and ultimately with the microscopic view of the viral particle.**

**Integration of data ( genetic, proteomic, microscopy)  is essential to observe typical or atypical viral behaviour.**

**Minor suggestions:**

 Request citation and clarity on the statement - “several studies have reported….intestinal bacterial co-infection in COVID-19 patients”.

**R : we have added the references 9-11 (line 20) : **

- **Yeoh YK, Zuo T, Lui GC, Zhang F, Liu Q, Li AY, Chung AC, Cheung CP, Tso EY, Fung KS, Chan V, Ling L, Joynt G, Hui DS, Chow KM, Ng SSS, Li TC, Ng RW, Yip TC, Wong GL, Chan FK, Wong CK, Chan PK, Ng SC. Gut microbiota composition reflects disease severity and dysfunctional immune responses in patients with COVID-19. Gut. 2021 Apr;70(4):698-706. doi: 10.1136/gutjnl-2020-323020. Epub 2021 Jan 11. PMID: 33431578; PMCID: PMC7804842;**
- **Yamamoto S, Saito M, Tamura A, Prawisuda D, Mizutani T, Yotsuyanagi H. The human microbiome and COVID-19: A systematic review. PLoS One. 2021 Jun 23;16(6):e0253293. doi: 10.1371/journal.pone.0253293. PMID: 34161373; PMCID: PMC8221462 ; **
- **Wang, B., Zhang, L., Wang, Y. et al. Alterations in microbiota of patients with COVID-19: potential mechanisms and therapeutic interventions. Sig Transduct Target Ther 7, 143 (2022). https://doi.org/10.1038/s41392-022-00986-0)**

Request citation and clarity on the statement - “Observations of possible links between the animal gut microbial environment and coronaviruses have been reported over time”. The current citations do not have evidence for a possible link to the gut microbial environment.

**R : We have refreshed the final part of the phrases in : « *in some studies*,» and we have added the references 16-20 (Line 31): **

- **Li HY, Li BX, Liang QQ, Jin XH, Tang L, Ding QW, Wang ZX, Wei ZY (2020) Porcine deltacoronavirus infection alters bacterial communities in the colon and feces of neonatal piglets. Microbiologyopen 9:e1036 ; **
- **Felten S, Klein-Richers U, Unterer S, Bergmann M, Leutenegger CM, Pantchev N, Balzer J, Zablotski Y, Hofmann-Lehmann R, Hartmann K. Role of Feline Coronavirus as Contributor to Diarrhea in Cats from Breeding Catteries. Viruses. 2022 Apr 21;14(5):858. doi: 10.3390/v14050858. PMID: 35632600; PMCID: PMC9143444 ;**
- ** Meazzi S, Stranieri A, Lauzi S, Bonsembiante F, Ferro S, Paltrinieri S, Giordano A. Feline gut microbiota composition in association with feline coronavirus infection : A pilot study. Res Vet Sci. 2019 Aug ;125 :272-278. Doi : 10.1016/j.rvsc.2019.07.003. Epub 2019 Jul 9. PMID : 31326703 ; PMCID : PMC7111766. ; **
- **Storz J, Lin X, Purdy CW, Chouljenko VN, Kousoulas KG, Enright FM, Gilmore WC, Briggs RE, Loan RW. Coronavirus and Pasteurella infections in bovine shipping fever pneumonia and Evans' criteria for causation. J Clin Microbiol. 2000 Sep;38(9):3291-8. doi: 10.1128/JCM.38.9.3291-3298.2000. PMID: 10970373; PMCID: PMC87376; **
- **Saif LJ. Bovine respiratory coronavirus. Vet Clin North Am Food Anim Pract. 2010 Jul;26(2):349-64. doi: 10.1016/j.cvfa.2010.04.005. PMID: 20619189; PMCID: PMC4094360)**

Fig 1 reports “Viral load” although what is actually reported is the viral RNA concentration, which can be different from viral load because not all viral particles are lysed and provide RNA for detection, and some viral RNA is reported to come from non-viral reservoirs4.

**R : We have nothing to add to this reviewer's comment because we are in perfect agreement. The authors of reference 4 (Alexandersen et al.), clarify that nasopharyngeal samples contain subgenomic sequences of SARS-CoV-2. Textually they write: *'The results described here fully support that SARS-CoV-2 genomic and subgenomic RNAs are present in diagnostic samples even in late infection/after active infection' and 'The detection of subgenomic RNA is therefore not direct evidence of active infection instead its presence at lower levels than virion genomic RNA results in detection for a shorter period of time unless using, e.g., highly sensitive NGS*'.**

**Similarly, in the tables presented in figure 1, we do not want to indicate who among the family members in the presented case series is infectious or not because the story in the paper describes well that they are all asymptomatic, except for the little girl.  In the paper, we never speculate on the correlation between viral charge and high infectivity. The tables only show how with the passage of a fairly long time (figure 1 C and D), 60 days, the presence of traces of viral RNA is no longer detected, in agreement with many contemporary studies. It should also be pointed out that reference 4, Alexandersen et al., describes the nasopharyngeal sampling as a viral RNA collection event, and readers and scientists should reflect that the sampling is performed on mucous membranes that are overabundant with bacteria and that it cannot be excluded that subgenomic sequences in non-SARS-CoV-2 sufferers could have bacteria, also, as a reservoir.**

**We do not associate the viral load to the disease state, nor do we speculate on high viral replication and high infectivity. Viral load is now a usual term used in scientific work, and the accuracy of the reviewer is relevant, but at this point, should specify the wording of many of the works currently published. However, we corrected everywhere the legend of figure 1 and other phrases in “ *RNA viral Concentration*.”**

 The manuscript calls for major editorial revisions given numerous improper usages of phrases. For instance - a) There are no “fecal-oral bacteria”, which suggests specific bacteria in the feces are able to be transmitted orally. b) It is unclear what “Bacterial feces” means. c) “RNA viral load count” appears miswritten.

**R : a) we apologize to the reviewer. We want to say bacteria from the gastrointestinal tract, and we replaced the phrase with " *bacteria of the human gut microbiome.*"**

**b) The sentence was rewritten: « *The* *feces of this patient was cultured in bacterial culture media and after 30 days, the pellet of bacteria* *, have been analyzed by TEM, immune-EM, and by fluorescence microscopy, and a set of obtained images is shown in* *Figure 2* *( for more details see supplementary material-s.m.)* *».* Line 133-136**

**c) The word count is a typo, and we followed the reviewer’s suggestion and pointed out that it is RNA concentration**

**Additional notes:**

 I don’t have experience with interpreting TEM images and therefore leave this comment here rather than as a cause for major revision. TEM images are complex requiring the appropriate collection and processing of samples, the inclusion of controls, and careful interpretation of observations. The improper use of EM to study SARS-CoV-2 from tissue samples has been a cause for concern5,6. To my untrained eye, Fig 2 seems to be another example of misinterpreted EM data because - 

 If Fig 2a in fact displays SARS-CoV-2 viral particles inside a bacterium, it is surprising to me that the bacterial host appears to be entirely filled by viral particles with no room for its essential, native molecules. To me, this figure seems like that of the widely described multivesicular bodies that are unrelated to SARS-CoV-2 and yet regularly misinterpreted6.  

 The figures don’t appear to have sufficient resolution to highlight the ultrastructures typical of SARS-CoV-2 that are required to unmistakable identify viral particles5.

 If Fig 2b in fact presents the case of a bacteria with viral particles around it, it merely appears like viral particles proximal to the bacteria in this image, with no evidence that these viruses in fact infected the bacteria. The conclusion that viral particles are found in the extracellular matrix of gut bacteria is an over-reach without sufficient evidence.

**R: We thank the reviewer for finally raising the same issue as the public comment to the present manuscript left by Dr.Michael Laue. We will disregard the fact that he states that he is not an expert in electron microscopy but we respectfully respond to his objections:**

**The article by Dittmayer and Laue et al. (5) that both we cite in the text of the manuscript and the reviewer cites in support of the misinterpretations that can be made during sample processing and during the interpretation of viral particles should be clarified that the authors emphasize the importance of matching, i.e., associating the EM image with genetics as well, attesting to the presence of the viral pathogen of interest or vice-versa. They are the ones who make it clear that imaging must be supported by genetic or protein testing, and in fact, they quote exactly that: “… *the presence of SARS-CoV-2 particles (figure A-D *EM *) complements the molecular traces of SARS-CoV-2s pecific proteins or nucleic acids*”. Our study demonstrated exactly that: EM images of virus-like particles combined with genetic confirmation of the presence of SARS-CoV-2, immunofluorescence microscopy with antibodies, and other tests, now described in the supplementary materials (s.m.).  The addition of immunofluorescence images highlighting both GRAM+ bacteria and fluorescence of the nucleocapsid protein of SARS-CoV-2 is also important. However, as a final note, we have added in the supplementary materials (s.m.) many other evidences, including that of nitrogen isotope, added to the culture medium, which marks the proteins of SARS-CoV-2 (2) and lays a stable stone to our experiments.**

**With due respect to the reviewer, it should be noted that citation #6 (Calomeni E. et al.), taking as a reference to highlight how SARS-CoV-2 particles can be confused with multivesicular bodies, states the following: " *MVBs were always been identified in podocytes (1 to 4 podocytes per glomerulus), but we have not seen them in tubular epithelial cells. MVBs were occasionally observed in endothelial cells (mainly arterial or arteriolar) and in a parietal glomerular epithelial cell of biopsy.***

***MVBs theoretically may represent podocyte endocytosis with subsequent formation of intracytoplasmic microvesicles resembling viruses." ......" However, microvesicles are commonly "free-floating" in the cytoplasm of many cell types, including tubular epithelial cells."............" caution is suggested when identifying a virus by EM in tissue sections."***

**In other words, MVBs are typical formations in animal tissues, not found in bacterial cultures. They cannot be confused with viral particles that we show in bacterial cultures even light-years away. They are by no definition at all associable with the viral particles we show in Figure 2A and 2B. They are completely different situations and two different substrates so there is really no basis for confusing the viral particles we show with the MVB.**

**It was made clear in the text that the images are from a 30-day culture of prokaryotic cells, bacteria, and that there are no mammalian cells; furthermore, Figure 2B, even for a non-expert in microscopy, cannot be misunderstood as it is an image showing a bacterial lysis phase with viral particles around it. In the work of Bullock et al., (13) cited in the comment by Dr. Laue just in Figures 1C and 2C, are clearly visible viral particles indicated by the authors as coronavirus particles perfectly identical to those we show inside the bacterium in our Figure 2A and around the lysed bacterium in Figure 2B. Moreover, the authors cite the following sentence to demonstrate that *the protein spike, unless a special preparation with tannic acid is used, is almost never visible in microscopic images as they report:" (ii) Coronaviruses do have projections on the surface; however, in thin sections, the "spikes" on the outside are not always (indeed, not usually) clearly visible, unless specially stained (e.g., with tannic acid). They may or may not appear as a very short 'fuzz.*'" The ribonucleoprotein, with all due respect and contrary to Dr. Laue's comments, is clearly visible, just like that shown in the works of Dittmayer, Laue, et al. (5), and Bullock et al. (13).**

**The Aurion particles of 10 nm may overlap, but in the supplementary materials (s.m.), we show Figure 2s where the same test was repeated with the pre-ebbending technique in another facility and also satisfies this aspect challenged in the comments. (line 176- 194s.m.).**

**With due respect to the reviewer, all supporting data, including those added in the supplementary materials, dispel doubts shown, and it is still worth noting that the evidence of the nitrogen radioisotope found in the viral proteins after adding the element in the 30-day bacterial culture dispels all doubt. The data should be integrated and analyzed together, and not considered individually.**

 The figure is missing controls such as stained and unstained images of comparable bacteria without the presence of the alleged SARS-CoV-2 viral particles. Given these observations and the importance of this figure to the central tenet of this manuscript, I recommend the editorial team seeks input from an expert in TEM.

**R : In Image 3, we added panels F and G with only controls on gram+ bacteria in the absence of SARS-CoV-2. The objections made by the reviewer seem identical to those in the commentary by Dr. Michael Laue, an imaging expert. Responding to the reviewer, we also questioned Dr. Michael Laue's objections.**

**We remind the reviewer that these are the first images of coronavirus in bacterial cultures. There are no other comparisons in the current literature, so any speculation on the present manuscript finds as debate the serious shortcoming of the failure to control infectivity first on prokaryotic cells, which are much more abundant above the epithelial layer where virus-receptor interaction takes place.  What should be done before proclaiming for certain and establishing some possible pathogenetic mechanisms of infectivity, i.e., some possible viral protein and epithelial cell receptor binding interactions, is the control between coronaviruses in particular but also other RNA viruses in general and bacterial cells, much more abundant, it bears repeating, within the human microbiome. Moreover, even if an interaction does not take place, control also over bacterial metabolism and what they produce (2,14) in the presence of a novel viral pathogen should in no way be excluded. This, too, is a serious deficiency in possible controls.**

 The hypothesis that SARS-CoV-2 may infect bacteria is both biologically fascinating and clinically highly relevant.

**R: We apologize to the reviewer: but with due respect: it is no longer a hypothesis but new  observations (ref 2,14, 15).**

**We thank the reviewer for giving us an opportunity to engage in constructive scientific debate and to improve the present paper by highlighting what he thought was right and by improving what he thought was appropriate.**

 This is because there are no examples that I know of or can find of viruses that affect humans that can also retain the molecular machinery to affect bacterial cells. Therefore, the idea that this is possible is biologically fascinating and can pave the path to many follow-up studies. Further, SARS-CoV-2 continues to be a major threat, having claimed over 6 million people worldwide. Therefore, careful evaluation of the potential sources of future outbreaks is critical from a personal and public health standpoint. If in fact bacteria in the gut can harbor infectious SARS-CoV-2 viruses, this adds to another dimension of the COVID-19 pandemic that calls for urgent precautionary measures. Unfortunately, the current manuscript is missing many controls and methodological details, and has insufficient data to make the case that bacteria are in fact infected by SARS-CoV-2. Given the hugely significant impact that concluding gut bacteria can be infected by SARS-CoV-2 through a bacteriophage-like property can have to humanity, I encourage the authors and editorial team to exercise caution and responsibility in promoting this conclusion with insufficient evidence.

**R : We believe that the reviewer now has much more clarification, but may notice more information about the concepts laid out and the importance of doing the checks between viruses and bacteria, to avoid epidemiologically and pathogenic misdiagnoses in the article under review also on F1000--Petrillo M, Querci M, Brogna C *et al.* Evidence of SARS-CoV-2 bacteriophage potential in human gut microbiota [version 1; peer review: awaiting peer review]. *F1000Research* 2022, 11:292 ( https://doi.org/10.12688/f1000research.109236.1)**

 References

1. Petrillo M, Brogna C, Cristoni S, Querci M, et al.: Increase of SARS-CoV-2 RNA load in faecal samples prompts for rethinking of SARS-CoV-2 biology and COVID-19 epidemiology.F1000Res. 2021; 10: 370 PubMed Abstract | Publisher Full Text
2.  Brogna C, Brogna B, Bisaccia DR, Lauritano F, et al.: Could SARS-CoV-2 Have Bacteriophage Behavior or Induce the Activity of Other Bacteriophages?. Vaccines (Basel). 2022; 10 (5). PubMed Abstract | Publisher Full Text
3.  Pedersen R, Tornby D, Bang L, Madsen L, et al.: Rectally shed SARS-CoV-2 lacks infectivity: time to rethink faecal–oral transmission?. Nature Reviews Gastroenterology & Hepatology. 2021; 18 (9). Publisher Full Text
4. Alexandersen S, Chamings A, Bhatta T: SARS-CoV-2 genomic and subgenomic RNAs in diagnostic samples are not an indicator of active replication. Nature Communications. 2020; 11 (1). Publisher Full Text
5. Dittmayer C, Meinhardt J, Radbruch H, Radke J, et al.: Why misinterpretation of electron micrographs in SARS-CoV-2-infected tissue goes viral. The Lancet. 2020; 396 (10260): e64-e65 Publisher Full Text
6. Calomeni E, Satoskar A, Ayoub I, Brodsky S, et al.: Multivesicular bodies mimicking SARS-CoV-2 in patients without COVID-19. Kidney International. 2020; 98 (1): 233-234 Publisher Full Text
7. **Guo M, Tao W, Flavell RA, Zhu S. Potential intestinal infection and faecal-oral transmission of SARS-CoV-2. Nat. Rev. Gastroenterol. Hepatol. 2021;18:269-283. doi: 10.1038/s41575-021-00416-6.**
8. **Guo M, Tao W, Flavell RA, Zhu S. Reply to: Rectally shed SARS-CoV-2 lacks infectivity: time to rethink faecal-oral transmission? Nat Rev Gastroenterol Hepatol. 2021 Sep;18(9):669-670. doi: 10.1038/s41575-021-00503-8. PMID: 34312525; PMCID: PMC8311630**
9. **Zhou, P., Yang, XL., Wang, XG. et al. A pneumonia outbreak associated with a new coronavirus of probable bat origin. Nature 579, 270-273 (2020). https://doi.org/10.1038/s41586-020-2012-7**
10. **Stone R. A new killer virus in China? Science. (2014). Available online at: https://www.sciencemag.org/news/2014/03/new-killer-virus-china**
11. Floridia, M. & Cristoni, S. PROSAD: A powerful platform for instrument calibration and quantification. Rapid Commun. Mass Spectrom. 2014. doi: https://doi.org/10.1002/rcm.6808.): ARB= [Σ(SI*NF*COF)/ Σ (NF*COF)] where SI: Signal intensity converted to Counts/s; NF: Noise Factor; COF: Correction Factor
12. **Dunbar SA. Applications of Luminex xMAP technology for rapid, high-throughput multiplexed nucleic acid detection. Clin Chim Acta. 2006. doi: https://10.1016/j.cccn.2005.06.023**
13. **Bullock HA, Goldsmith CS, Miller SE. Best practices for correctly identifying coronavirus by transmission electron microscopy. Kidney Int. 2021 Apr;99(4):824-827. doi: 10.1016/j.kint.2021.01.004. Epub 2021 Jan 22. PMID: 33493525; PMCID: PMC7825881**
14. **Brogna C, Cristoni S, Petrillo M, Querci M, Piazza O, Van den Eede G. Toxin-like peptides in plasma, urine and faecal samples from COVID-19 patients. *F1000Res*. 2021;10:550. Published 2021 Jul 8. doi:10.12688/f1000research.54306.2**
15. **Petrillo M, Querci M, Brogna C *et al.* Evidence of SARS-CoV-2 bacteriophage potential in human gut microbiota [version 1; peer review: awaiting peer review]. F1000Research 2022, 11:292 ( https://doi.org/10.12688/f1000research.109236.1)**

# Reviewer report: Reviewer response for version 1

**DOI:** 10.5256/f1000research.81407.r125777

**Published:** 2022-03-07

**Authors:** Debojyoti Dhar

**Affiliations:** 1 Leucine Rich Bio Pvt. Ltd, Bengaluru, Karnataka, India

**Reviewer comments –**

1. The Figure 1B legend needs to be properly written or displayed.
2. Figure 1C, correct the spelling of culture as in Bacteria culture (legend).
3. In Table 1, data pertaining to the nasopharyngeal swab test need to be presented for all the subjects.
4. “At day 30 of bacterial culture of feces patient am1, the Luminex molecular test confirmed the presence of SARS-CoV-2 and the RNA viral load count was increased from 24 arbitrary unit (AU) (initial) to 520 AU (Final) (Figure 1B)...” – The figure description needs to be better (Figure 1B). The authors should better describe whose fecal sample was used in the culture in the figure description for 1B.
5. “Bacterial feces of this patient, after 30 days of bacterial culture, have been analyzed by TEM, immune-EM, and by fluorescence microscopy, and a set of obtained images is shown...” – Instead of bacterial feces, change to feces of this patient was cultured in bacterial culture media.
6. Figure 2D is not clear to this reviewer.
7. “In addition, the RNA virus could be present in the 48,1% of patients who were negative to OP/NP swab tests until 33 days.” – Please correct the % depiction.
8. “Here, we report the case of a symptomatic child for COVID 19, brought to her by one of the parents, whose family members had negative results with rapid antigen nasopharyngeal swab test.” – Please correct the grammatical error.
9. This reviewer has reservation on the “fecal-oral” transmission route being used by the authors. This paper and the earlier paper on this topic by the authors showed possible replication of the SARS-Cov2 virus like particles in bacterial culture. But neither observations prove the route of the viruses coming into the feces. As far as this reviewer is concerned, we still do not know how the virus gets to the gastrointestinal tract. So, instead of “fecal-oral” bacteria, gastrointestinal bacteria may be written.
10. “This surprising finding allows us to better clarify the first fecal-oral transmission of the virus and clearly shows that the reservoir of the virus is neither adults nor children but simply bacteria.” – This reviewer does not agree with this statement especially the second half of it.

Overall, this is an interesting finding and corroborates the earlier report of the same author (F1000Res. 2021 May 11; 10:370) about the presence of the SARS-Cov2 like virus particles in bacterial culture. This report is an advancement as some evidence of the presence of the virus like particles have been shown using TEM, immune-fluorescence microscopy. The presence of SARS-Cov2 in feces has been documented before and as such this report does not add anything new to this however what is interesting is the replication potential of the virus in bacterial culture. The authors might want to provide more assays and evidence to showcase the “phage-like” activity of the SARS-Cov2 as they propose.

 Finally, please crosscheck the text. Lots of grammatical and contextual errors are found, some of which have been highlighted above.

Are enough details provided of any physical examination and diagnostic tests, treatment given and outcomes?

Partly

Is the background of the cases’ history and progression described in sufficient detail?

Partly

Is sufficient discussion included of the importance of the findings and their relevance to future understanding of disease processes, diagnosis or treatment?

No

Is the conclusion balanced and justified on the basis of the findings?

Partly

Reviewer Expertise:

gut microbiome

I confirm that I have read this submission and believe that I have an appropriate level of expertise to confirm that it is of an acceptable scientific standard, however I have significant reservations, as outlined above.

## References

[1] : Increase of SARS-CoV-2 RNA load in faecal samples prompts for rethinking of SARS-CoV-2 biology and COVID-19 epidemiology. *F1000Res* .2021;10: 10.12688/f1000research.52540.3 370 10.12688/f1000research.52540.3 34336189 PMC8283343

# Response

**Published:** 2022-03-10

**Authors:** Carlo Brogna

**Affiliations:** Craniomed group facility, Italy

Dear Dr. Debojyoti Dhar,

 Thanks a lot for your valuable comments and suggestions that you have provided in the report.

 We will address all of them, together with those of other reviewers, in order to provide a fully revised version of the manuscript.

 Best regards,

 Carlo Brogna, on behalf of the authors.

# Response

**Published:** 2022-07-17

**Authors:** Carlo Brogna

**Affiliations:** Craniomed group facility, Italy

**Reviewer comments** **and Brogna et al. responses (R.)**

**First of all, we apologize to the reviewer for responding only now, but we also waited for the opinion of a second reviewer who arrived in early July so that we could review the manuscript only once.**

1. The Figure 1B legend needs to be properly written or displayed.

**R: the legend has been replaced in the following form: “ *This is the positive control of patient am1's bacteria derived from a stool sample, after 30 days of bacterial culture using our previously published method (21), performed with the Luminex molecular assay. The molecular assay reported a viral RNA concentration growth of up to 520 AU (arbitrary unit).”* Line 94-99**

1. Figure 1C, correct the spelling of culture as in Bacteria culture (legend).

**R: Done. Line 97**

  

1. In Table 1, data pertaining to the nasopharyngeal swab test need to be presented for all the subjects.

**R: We have added again the corrected table 1, in which there is the column with the data request.**

  

1. “At day 30 of bacterial culture of feces patient am1, the Luminex molecular test confirmed the presence of SARS-CoV-2 and the RNA viral load count was increased from 24 arbitrary unit (AU) (initial) to 520 AU (Final) (Figure 1B)...” – The figure description needs to be better (Figure 1B). The authors should better describe whose fecal sample was used in the culture in the figure description for 1B.

**Done. Line 94-99**

  

1. “Bacterial feces of this patient, after 30 days of bacterial culture, have been analyzed by TEM, immune-EM, and by fluorescence microscopy, and a set of obtained images is shown...” – Instead of bacterial feces, change to feces of this patient was cultured in bacterial culture media.

**R: We have changed as suggested in: “ *The* *feces of this patient was cultured in bacterial culture and after 30 days, the pellet of bacteria*…..” Line 133-137**

1. Figure 2D is not clear to this reviewer.

**R: We apologize with the reviewer and have clarified our mistake in the legend, describing figure 2D: *“* *(D)* *negative control of bacterial stool culture* *of a healthy person after 30 days, without primary antibody with only the secondary antibody.” *Line 174-176**

  

1.  “In addition, the RNA virus could be present in the 48,1% of patients who were negative to OP/NP swab tests until 33 days.” – Please correct the % depiction.

**R: We apologize to the reviewer and thank him for pointing this out to us. There was an error in bibliography #48, which we have replaced correctly, and where the proposed data are now visible. For clarity, we rephrase the sentence in the text as follows:**

**" *In a Systematic Review and Meta-analysis, at the beginning of the pandemic, it was observed that viral RNA was present in the stool in 48,1% of patients during the disease and that 70,3 % of patients had prolonged shedding that could extend beyond 33 days from the onset of the disease."* (2 here, 48in the manuscript). Line 264-267**

 8, “Here, we report the case of a symptomatic child for COVID 19, brought to her by one of the parents, whose family members had negative results with rapid antigen nasopharyngeal swab test.” – Please correct the grammatical error.

**R: We replaced it with: “ *Here we report the case of a child symptomatic for COVID 19, transmitted by one of the parents, whose relatives had tested negative* *on the rapid antigenic nasopharyngeal swab test*.” Line 2826-287**

 This reviewer has reservation on the “fecal-oral” transmission route being used by the authors. This paper and the earlier paper on this topic by the authors showed possible replication of the SARS-Cov2 virus like particles in bacterial culture. But neither observations prove the route of the viruses coming into the feces. As far as this reviewer is concerned, we still do not know how the virus gets to the gastrointestinal tract. So, instead of “fecal-oral” bacteria, gastrointestinal bacteria may be written.

**R: We appreciate the reviewer's critical viewpoint. In our recent paper published in ref. 3, we supported this possible additional transmission pathway. Still, the reviewer's suggestion is very cautious, and in agreement with him, we have chosen to replace the term fecal-oral bacteria with” *bacteria of the human gut microbiome*”. We hope he agrees.**

 “This surprising finding allows us to better clarify the first fecal-oral transmission of the virus and clearly shows that the reservoir of the virus is neither adults nor children but simply bacteria.” – This reviewer does not agree with this statement especially the secondhalf of it.

**R: With all due respect to the reviewer and his valuable help in improving this article, let us argue our opinion in a few lines. If we consider how many sites report the presence of viral RNA in wastewater (4-6), as described in the current literature, although everyone knows that RNA by definition is an easily degradable (7) molecule, and if we consider how the first six miners in the Yunnah caves in China, in close contact with bat guano (feces), became ill with CORONAVIRUS RATG13 pneumonia (8-9), and furthermore if we also consider our recent work Brogna et al. (3). where we performed many other tests, we can more confidently assert that bacteria *could *represent a *potential reservoir* of the virus and, as in the work cited above, we argued that it could behave as both a lytic and lysogenic bacteriophage. However, with the caution suggested by the reviewer, we have changed the last sentence to: “ *This first observation invites us to pay more attention to the fecal-oral transmission route of the virus and suggests as a further possible reservoir of the virus also the bacteria of the human gut microbiome.” *Line 298-300**

 Overall, this is an interesting finding and corroborates the earlier report of the same author (F1000Res. 2021 May 11; 10:370) about the presence of the SARS-Cov2 like virus particles in bacterial culture. This report is an advancement as some evidence of the presence of the virus like particles have been shown using TEM, immune-fluorescence microscopy. The presence of SARS-Cov2 in feces has been documented before and as such this report does not add anything new to this however what is interesting is the replication potential of the virus in bacterial culture. The authors might want to provide more assays and evidence to showcase the “phage-like” activity of the SARS-Cov2 as they propose.

**R: We can now reassure the reviewer on this point because the much-required evidence has been made public in the publications of Brogna et al. and Petrillo et al. (3,10), and we added some of the other results in the supplementary materials in which we have added evidence of nitrogen isotopic labeling also in viral particle proteins..**

**The reviewer's comments and corrections were invaluable in improving this work. We thank them for making the work solid.**

 Finally, please crosscheck the text. Lots of grammatical and contextual errors are found, some of which have been highlighted above.

 The work was reviewed by a native speaker.
